# Population genomic analysis provides evidence of the past success and future potential of South China tiger captive conservation

**DOI:** 10.1186/s12915-023-01552-y

**Published:** 2023-04-18

**Authors:** Chen Wang, Dong-Dong Wu, Yao-Hua Yuan, Meng-Cheng Yao, Jian-Lin Han, Ya-Jiang Wu, Fen Shan, Wan-Ping Li, Jun-Qiong Zhai, Mian Huang, Shi-Ming Peng, Qin-Hui Cai, Jian-Yi Yu, Qun-Xiu Liu, Zhao-Yang Liu, Lin-Xiang Li, Ming-Sheng Teng, Wei Huang, Jun-Ying Zhou, Chi Zhang, Wu Chen, Xiao-Long Tu

**Affiliations:** 1 Guangzhou Zoo & Guangzhou Wildlife Research Center, Guangzhou, 510070 China; 2 State Key Laboratory of Genetic Resources and Evolution, Kunming Institute of Zoology, Chinese Academy of Sciences, Kunming, 650201 China; 3 Kunming Natural History Museum of Zoology, Kunming Institute of Zoology, Chinese Academy of Sciences, Kunming, 650223 Yunnan China; 4 Kunming College of Life Science, University of the Chinese Academy of Sciences, Kunming, 650204 China; 5 Shanghai Zoo, Shanghai, 200336 China; 6 CAAS-ILRI Joint Laboratory on Livestock and Forage Genetic Resources, Institute of Animal Science, Chinese Academy of Agricultural Sciences (CAAS), Beijing, 100193 China; 7 International Livestock Research Institute (ILRI), Nairobi, 00100 Kenya; 8 Wangcheng Park, Luoyang, 471000 China; 9 Suzhou Shangfangshan Forest Zoo, Suzhou, 215009 China; 10 Chongqing Zoo, Chongqing, 401326 China; 11 Nanchang Zoo, Nanchang, 330025 China; 12 Chinese Association of Zoological Gardens, Beijing, 100037 China; 13 Qinghai Province Key Laboratory of Crop Molecular Breeding, Key Laboratory of Adaptation and Evolution of Plateau Biota, Northwest Institute of Plateau Biology, Chinese Academy of Sciences, Xining, 810008 Qinghai China

**Keywords:** South China tiger, Chromosome-level genome, Whole genome sequencing, Genomic inbreeding, Deleterious mutations

## Abstract

**Background:**

Among six extant tiger subspecies, the South China tiger (*Panthera tigris amoyensis*) once was widely distributed but is now the rarest one and extinct in the wild. All living South China tigers are descendants of only two male and four female wild-caught tigers and they survive solely in zoos after 60 years of effective conservation efforts. Inbreeding depression and hybridization with other tiger subspecies were believed to have occurred within the small, captive South China tiger population. It is therefore urgently needed to examine the genomic landscape of existing genetic variation among the South China tigers.

**Results:**

In this study, we assembled a high-quality chromosome-level genome using long-read sequences and re-sequenced 29 high-depth genomes of the South China tigers. By combining and comparing our data with the other 40 genomes of six tiger subspecies, we identified two significantly differentiated genomic lineages among the South China tigers, which harbored some rare genetic variants introgressed from other tiger subspecies and thus maintained a moderate genetic diversity. We noticed that the South China tiger had higher *F*_ROH_ values for longer runs of homozygosity (ROH > 1 Mb), an indication of recent inbreeding/founder events. We also observed that the South China tiger had the least frequent homozygous genotypes of both high- and moderate-impact deleterious mutations, and lower mutation loads than both Amur and Sumatran tigers. Altogether, our analyses indicated an effective genetic purging of deleterious mutations in homozygous states from the South China tiger, following its population contraction with a controlled increase in inbreeding based on its pedigree records.

**Conclusions:**

The identification of two unique founder/genomic lineages coupled with active genetic purging of deleterious mutations in homozygous states and the genomic resources generated in our study pave the way for a genomics-informed conservation, following the real-time monitoring and rational exchange of reproductive South China tigers among zoos.

**Supplementary Information:**

The online version contains supplementary material available at 10.1186/s12915-023-01552-y.

## Background

The tiger (*Panthera tigris*) is one of the largest felids and a widely recognized flagship species of wildlife conservation in the world. There are six commonly accepted living tiger subspecies, including the South China tiger (*P. t. amoyensis*), Amur tiger (*P. t. altaica*), Indochinese tiger (*P. t. corbetti*), Malayan tiger (*P. t. jacksoni*), Bengal tiger (*P. t. tigris*), and Sumatran tiger (*P. t. sumatrae*) [1–3]. Among them, the Sumatran tiger is the only island population to be distinctive from all continental tigers and the Amur tiger splits last within mainland Asia [4], while the South China tiger is the rarest tiger subspecies [5]. In the 1950s, around 4000 South China tigers were found in 13 provinces in China [6]. Unfortunately, up to 3000 tigers were hunted as a pest and killed mercilessly during that period. Habitat loss/fragmentation further accelerated the decline of these tigers [7]. Only in 1979, the Chinese government banned the hunting of the tigers, whereas the number of the South China tiger was estimated to be only 30–80 in 1996. No South China tiger was directly sighted in the wild since the 1990s [7], it was therefore believed to be “functionally extinct” by many scientists, followed by an official declaration of its extinction in the wild in 2012.

Considerable efforts have been made to rescue the South China tiger through a captive breeding program in China [8]. Since 1955, a total of 27 male and 20 female South China tigers were kept in captivity according to the studbook [9]. In 1963, the first success of captive breeding of this tiger subspecies was achieved in the Guiyang Qianling Zoo. Detailed pedigree records indicated that all captive South China tigers were the descendants of two male and four female wild-caught tigers, which were managed in the Shanghai Zoo (one male and one female from Guizhou as well as one female from Fujian provinces) and Guiyang Qianling Zoo (one male and two females from Guizhou province) [9]. These descendants formed two founder lineages managed independently by the Shanghai and Guiyang Qianling Zoos over the 1970s. During 1972-1984, the captive population expanded quickly from 13 to 49 tigers. However, fewer cubs were born during 1985–1995 and the average survival rate of newborn cubs was low [10, 11], following the rapid loss of genetic variations and possible inbreeding depression within this small population.

The Chinese Association of Zoological Gardens commenced a coordinated South China tiger captive breeding and management program in 1994. From 1995, the South China Tiger Committee (renamed as Tiger Taxonomy Advisory Group, the Tiger TAG, in 2015) has been organizing an annual review of the captive population and designing breeding and exchange plans [12]. The Tiger TAG set a goal to maintain 90% of the genetic variations present in the captive population in 1995 and targeted to sustain 70% of the variations over the next 100 years. Since then, the breeding tigers began to be exchanged between the zoos based on their pedigree records and the number of the South China tigers increased rapidly. There were 205 South China tigers managed in captivity by 16 Chinese zoos and 18 individuals in the Laohu Valley Reserve in South Africa by 2020.

Because of the speculated inbreeding depression and extinction of the South China tiger in the wild, some experts suggested to introduce genetic supplementation from other tiger subspecies into the captive South China tiger population to enlarge its gene pool [8]. Hybridization between the South China and Amur tigers was believed to have occurred in some Chinese zoos [13], which was supported by genetic characterization using mitochondrial and microsatellite DNA markers [14, 15]. It is evident that one allele at individual genetic loci throughout the genomes of a small and managed population can be fixed rapidly, leading to an increase in genomic homozygosity and subsequently the inbreeding load [16, 17]. Considering the positive correlation between genetic heterozygosity and fitness [18, 19], it is urgently needed to examine the genomic landscape of existing genetic variations that have driven the past rescue of the South China tiger, in reference to large-scale population genomic studies on other tiger subspecies [1, 4, 20].

In this study, we assembled a de novo chromosome-level genome and re-sequenced 29 whole genomes of the captive South China tigers collected from four major zoos. Combined with other whole-genome data of six tiger subspecies, we characterized the genetic diversity, population genetic structure, demographic history, genomic inbreeding, and deleterious mutation load of the captive South China tiger population. These findings do not only explain the successful breeding history of the South China tigers in captivity, but also pave the way for a genomics-informed management by applying genome-wide markers to routinely monitor and sustain their critical genetic variations in the future.

## Results

### De novo genome assembly and whole-genome re-sequencing of the South China tigers

**Fig. 1:**
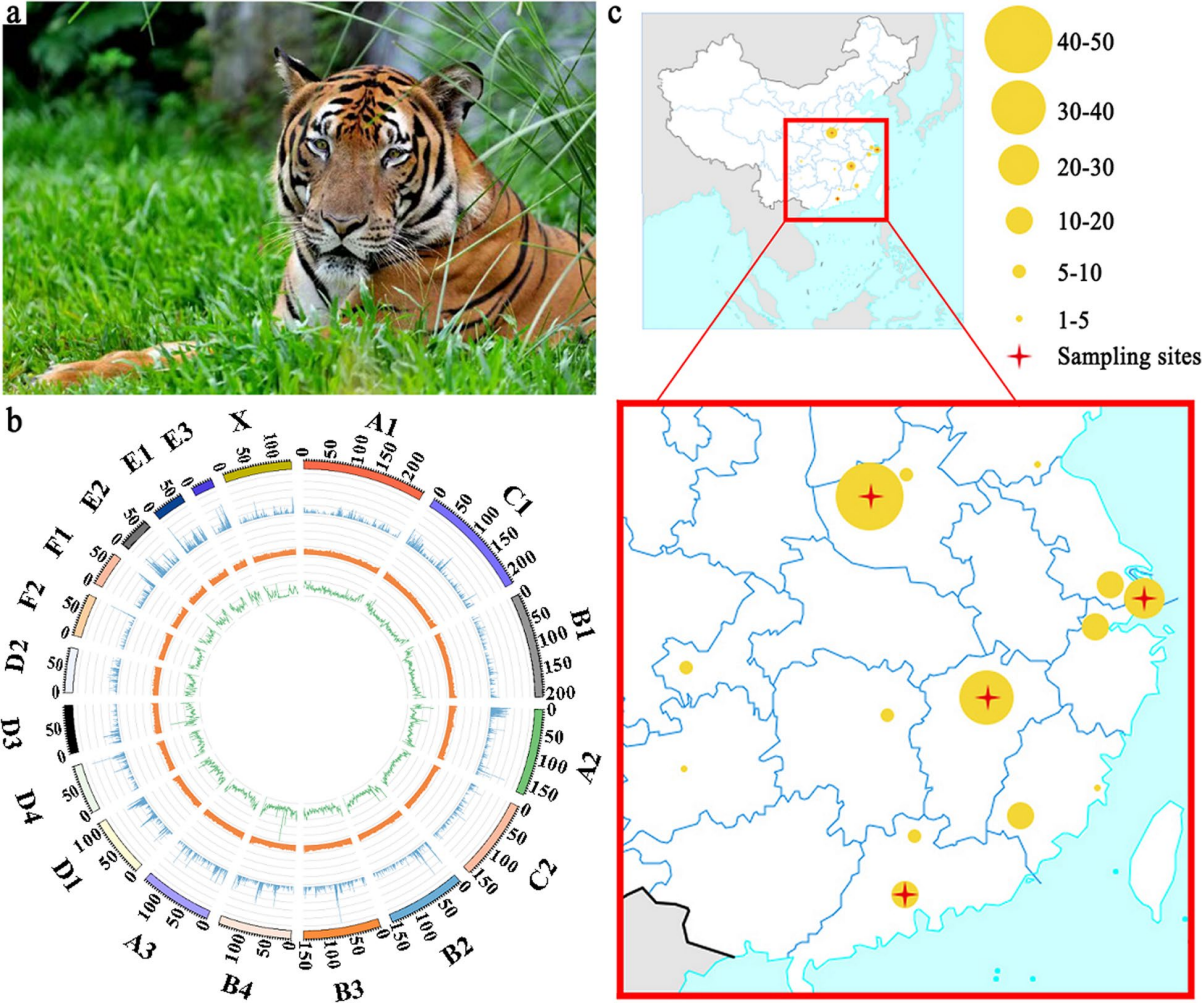
Genome of South China tiger. **a** A male South China tiger. **b** Circos plot of South China tiger genomic features. Outer to inner: pseudo-chromosome, gene density (500-kb window) (with higher gene density indicated by higher blue column), GC content (500-kb window), and SNP density (500-kb window). **c** Distribution of the captive South China tigers in China. Yellow circles show 15 city locations of the captive South China tigers, including Luoyang, Zhengzhou, Linyi, Suzhou, Shanghai, Hangzhou, Nanchang, Chongqing, Chengdu, Guiyang, Changsha, Fuzhou, Longyan, Shaoguan, and Guangzhou. Circle size is proportional to the number of South China tigers in each city. All data are from the South China tiger studbook (2020). Red cross represents the sampling site of the South China tigers in our study

To investigate the genetic variations of the South China tiger (Fig. 1a), we first constructed a high-quality de novo assembly of the South China tiger genome using a combination of high-fidelity short-read sequencing [21], long-read single-molecule real-time sequencing [22], optical mapping [23], and Hi-C [24] technologies. We generated 122.31 Gb (50.96×) of PacBio long reads, 1,011.73 Gb (421.55×) of Illumina paired-end short reads, 440.32 Gb (183.47×) of Bionano optical molecules, and 532.46 Gb (221.86×) of Hi-C data (Additional file 1: Tables S1 and S2). The K-mer [25] analysis revealed its genome size to be 2.47 Gb (Additional file 1: Table S3 and Additional file 2: Figure S1). After being polished with the PacBio long reads and corrected using the Illumina short reads [26], the PacBio-based initial assembly resulted in a contig N50 at 6.20 Mb. We scaffolded the PacBio contigs using the Bionano optical mapping data. The resulting scaffolds were further clustered into chromosome-scale scaffolds using the Hi-C data (Additional file 2: Figure S2). Finally, the de novo assembly yielded 2.44 Gb of genomic sequences with a contig N50 at 6.13 Mb and a scaffold N50 at 150.19 Mb (Fig. 1b and Additional file 1: Table S4). The de novo assembly contained 19 pseudo-chromosomes anchored with 2.40 Gb of contigs (99.35%) and 2.42 Gb of scaffolds (99.36%), showing a high collinearity with the domestic cat (*Felis catus*) reference genome (FelCat9.0, Ensembl release 98, last access in September 2019), except for E3 chromosome (Additional file 1: Table S5 and Additional file 2: Figure S3). In the analysis of complete Benchmarking Universal Single-Copy Orthologs (BUSCO), our assembled genome covered 95.5% of the BUSCO genes [27] (Additional file 1: Table S6). By integrating the homology- and de novo-based predictions, 20,908 protein-coding genes were annotated (Additional file 1: Tables S7-S9). There were 844.92 Mb (34.98% of the genome size) of repetitive elements as well as 568 microRNAs (miRNA), 6,309 transfer RNAs (tRNA), 993 ribosomal RNAs (rRNA), and 1410 small nuclear RNAs (snRNA) (Additional file 1: Table S10). Altogether, we assembled and annotated the South China tiger genome (Amotig1.0) [28] as one of the top-quality chromosome-level genomes of all big cats (Additional file 1: Table S4) [29–36].

To explore genetic variations in the captive South China tiger population, we re-sequenced the whole genomes of 29 South China tigers collected from four major zoos in China (Fig. 1c). A domestic cat was also re-sequenced and used as an outgroup. We generated around 1200 Gb of whole-genome sequencing data with an average coverage depth of 15.63× per genome [37]. We combined our data with the 40 published genomes of six tiger subspecies (10.38-29.59× coverage depths) [38, 39] (Additional file 1: Tables S11-S13 and Additional file 2: Figures S4 and S5). Altogether, we identified 10.21 million high-quality single nucleotide polymorphisms (SNPs) in these 69 tiger genomes after stringent quality control and alignment of all sequencing data against the Amotig1.0 genome (Additional file 1: Tables S14 and S15 and Additional file 2: Figure S6).

### Genetic variations among and demographic history of tiger subspecies

All tiger subspecies have experienced severe population bottlenecks due to human hunting and habitat loss/fragmentation, we thus compared the levels of their genetic variations. It was evident that the Sumatran tiger carried the least genetic diversity in terms of genome-wide heterozygosity and nucleotide diversity (π = 0.553 × 10^−3^) (Fig. 3a, Additional file 1: Table S16, and Additional file 2: Figures S7 and S8), but the highest genetic differentiation from other tiger subspecies (*F*_ST_ = 0.324–0.459) (Additional file 1: Table S17 and Additional file 2: Figure S9), confirming the observations of previous studies [1, 4]. However, the genetic diversity of the South China tiger was not as low (π = 0.657 × 10^−3^) (Additional file 1: Table S16 and Additional file 2: Figure S7) as what was inferred from its pedigree records [9], and the genomic heterozygosity of the South China tiger was moderate among six tiger subspecies (Additional file 1: Table S15 and Additional file 2: Figure S8). Nonetheless, the South China tiger also showed a significant genetic differentiation from other tiger subspecies (*F*_ST_ = 0.278–0.459) (Additional file 1: Table S17 and Additional file 2: Figure S9).

**Fig. 2:**
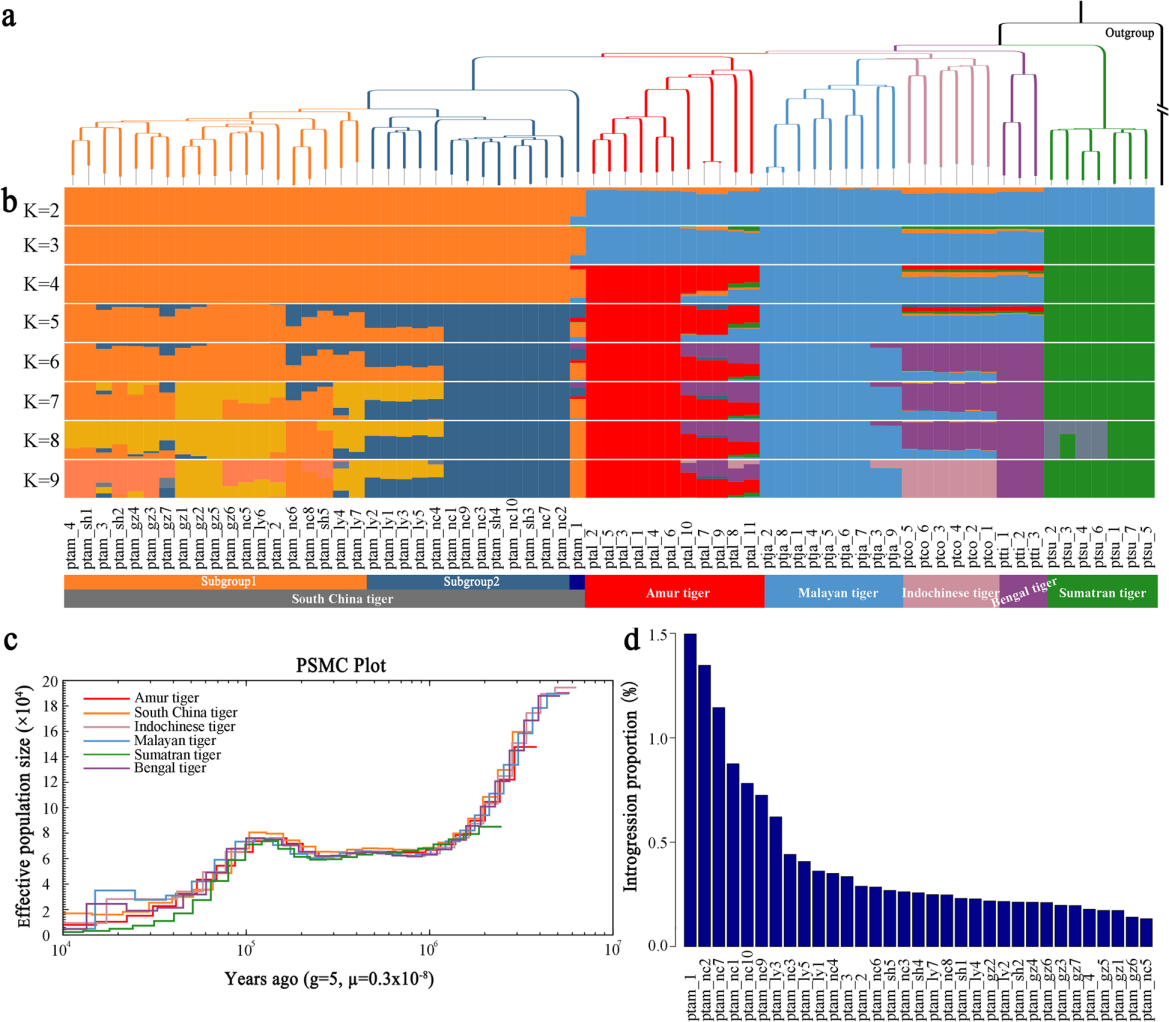
Genetic components of extant South China tigers. **a** Phylogenetic relationship of the South China tiger with other tiger subspecies, with a domestic cat as an outgroup, all nodes are 100% of reliability. **b** Population genetic structuring of different tiger subspecies. **c** PSMC plot of the inferred demographic histories of each tiger subspecies. **d** Average ratio of introgression between the South China tiger and other tiger subspecies

To clarify the phylogenetic relationships among six tiger subspecies, we performed neighbor-joining (NJ) phylogenetic reconstructions, principal component analysis (PCA), and a model-based ancestry estimation using ADMIXTURE software to infer their population genetic structure. We reconstructed the NJ trees based on pairwise genetic distances with the domestic cat as an outgroup, which supported the taxonomic status of six distinct tiger subspecies [1, 3, 40, 41] (Fig. 2a). The PCA (Additional file 2: Figure S10), maximum-likelihood tree (Additional file 2: Figure S11), and identity-by-state analysis (Additional file 2: Figures S12 and S13) all verified their phylogenetic relationships at the subspecies level. However, the South China tigers were further differentiated into two lineages (e.g., lineages 1 and 2), except one particular tiger labeled as the ptam_1 to be a potential hybrid, while the Amur, Sumatran, and Malayan tigers formed three additional genetic clusters at *K* = 5 (Fig. 2b). All six tiger subspecies were differentiated from each other at *K* = 9, despite potential gene flow among some tiger subspecies (Fig. 2b). Additionally, the population genetic structure of only captive South China tigers verified the two genomic lineages at *K* = 2 (Additional file 2: Figure S14), which mirrored the Shanghai and Guiyang founder lineages [10, 11]. Meanwhile, the pairwise sequential Markovian coalescent (PSMC) plots showed that all tigers experienced continuous bottlenecks since the onset of the Last Glacial Period (Fig. 2c and Additional file 2: Figure S15), a pattern that was observed in a previous study [1].

### Limited gene flow from other tiger subspecies into the captive South China tiger population

To detect a signal of potential genetic admixture/introgression from other tiger subspecies into the South China tiger population (e.g., the lineages 1 and 2 as well as the ptam_1 tiger), we applied several population genetic analyses, including the ABBA-BABA [42, 43], Dsuite [44], and TreeMix methods [45]. All results clearly indicated the highest admixture in the ptam_1 tiger [1] as a hybrid from the Indochinese tiger (Fig. 2b, d, and Additional file 2: Figures S16 and S20). Although genetic admixture occurred among tiger subspecies, there was limited gene flow from other tiger subspecies into the lineages 1 and 2 of the South China tiger (Additional file 1: Tables S16 and S17 and Additional file 2: Figures S17-S19). Altogether, we observed very limited introgression (0.13–1.50%) among the South China tiger genomes (Fig. 2d), indicating their genetic uniqueness to be warranted for a full protection.

### Genomic inbreeding among tiger subspecies

**Fig. 3:**
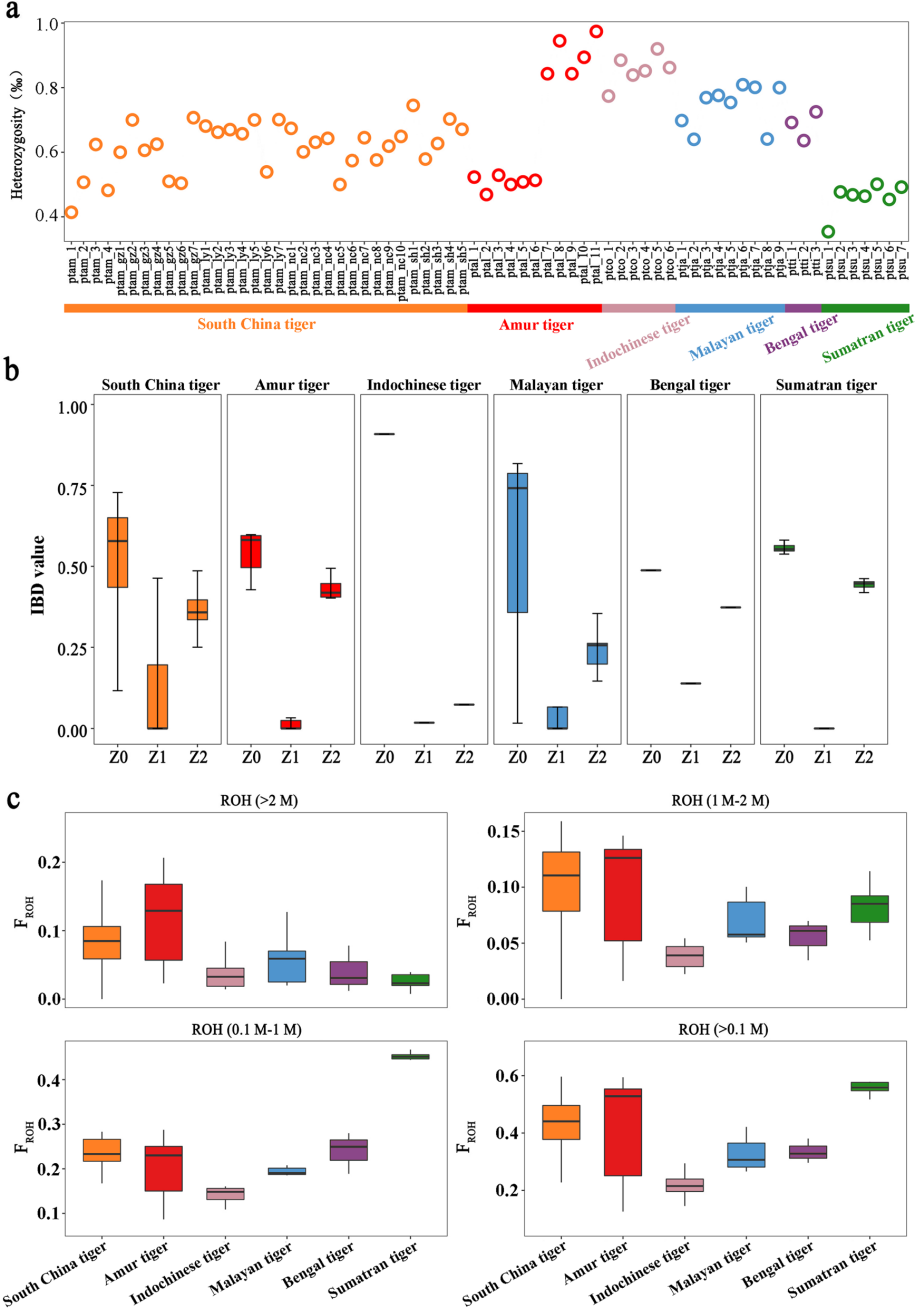
Comparison of genetic variations among different tiger subspecies. **a** Genome-wide heterozygosity per individual. **b** Pairwise relatedness based on allelic identity-by-descent (IBD), that is, genetic identity because of a recent common ancestor. Z0, Z1, and Z2 are the probabilities that two individuals share neither, one or two alleles IBD. **c** Genomic inbreeding coefficients (*F*_ROH_) based on different lengths of runs of homozygosity (ROH), with a minimum length of 100 kb

To characterize the speculated inbreeding depression among the South China tigers, we determined pairwise relatedness between individuals within each tiger subspecies using allelic identity-by-descent (IBD) [46] (Fig. 3b and Additional file 1: Table S20). Further, we calculated individual inbreeding based on genome-wide SNPs using the inbreeding coefficients *F*_H_ (a measure of the increase in individual SNP homozygosity compared with mean Hardy-Weinberg expected homozygosity) and *F*_ROH_ [47] (based on the runs of homozygosity (ROH) ≥ 100 kb). Among all tiger subspecies, the highest levels of the relatedness, *F*_H_, and *F*_ROH_ in ROH < 1 Mb were observed in the Sumatran tiger, however, its *F*_ROH_ values were reduced down to the lowest in ROH > 2 Mb, indicating the effects of strong founder and/or bottleneck events occurred in the recent past (Additional file 1: Table S21 and Additional file 2: Figure S21), while the Amur tiger seemed to have experienced constant inbreeding pressure as evidenced by their second highest relatedness, *F*_H_, and *F*_ROH_ values in ROH > 100 kb, which were increased to the highest in ROH > 2 Mb. On the other hand, the South China tigers shared a similar pattern of their relatedness as what was observed among other tiger subspecies, but they had relatively high *F*_ROH_ values in ROH > 100 kb and 1 Mb, compared with the Bengal, Indochinese, and Malayan tigers, which were likely the impact of recent inbreeding/founder events coupled with contraction of their effective population size (Fig. 3c). Among the four major zoos, the South China tigers in the Shanghai Zoo had the most number of ROH (average number = 3293, ROH = 100 kb–1 Mb), but only all the South China tigers in the Shanghai Zoo and the hybrid ptam_1 did not have any long ROH (> 2 Mb) (Additional file 1: Table S21), suggesting the captive tigers in the Shanghai Zoo to be least inbred. Furthermore, the South China tiger had the lowest *F*_ROH_ (average value = 0.33, ROH ≥ 100 kb) (Additional file 1: Table S21). Although the pedigree-based inbreeding coefficients (*F*_p_) among the South China tigers were as high as 0.1796–0.5048 [9], their relatedness was more strongly correlated with both *F*_H_ and *F*_ROH_ than *F*_p_ when high-density SNPs were available [48] (Additional file 2: Figure S22). The genome-wide SNP-based individual inbreeding estimates are thus recommended for assisting the decision-making of captive breeding of the South China tiger.

### Accumulation and purging of deleterious mutations among tiger subspecies

**Fig. 4:**
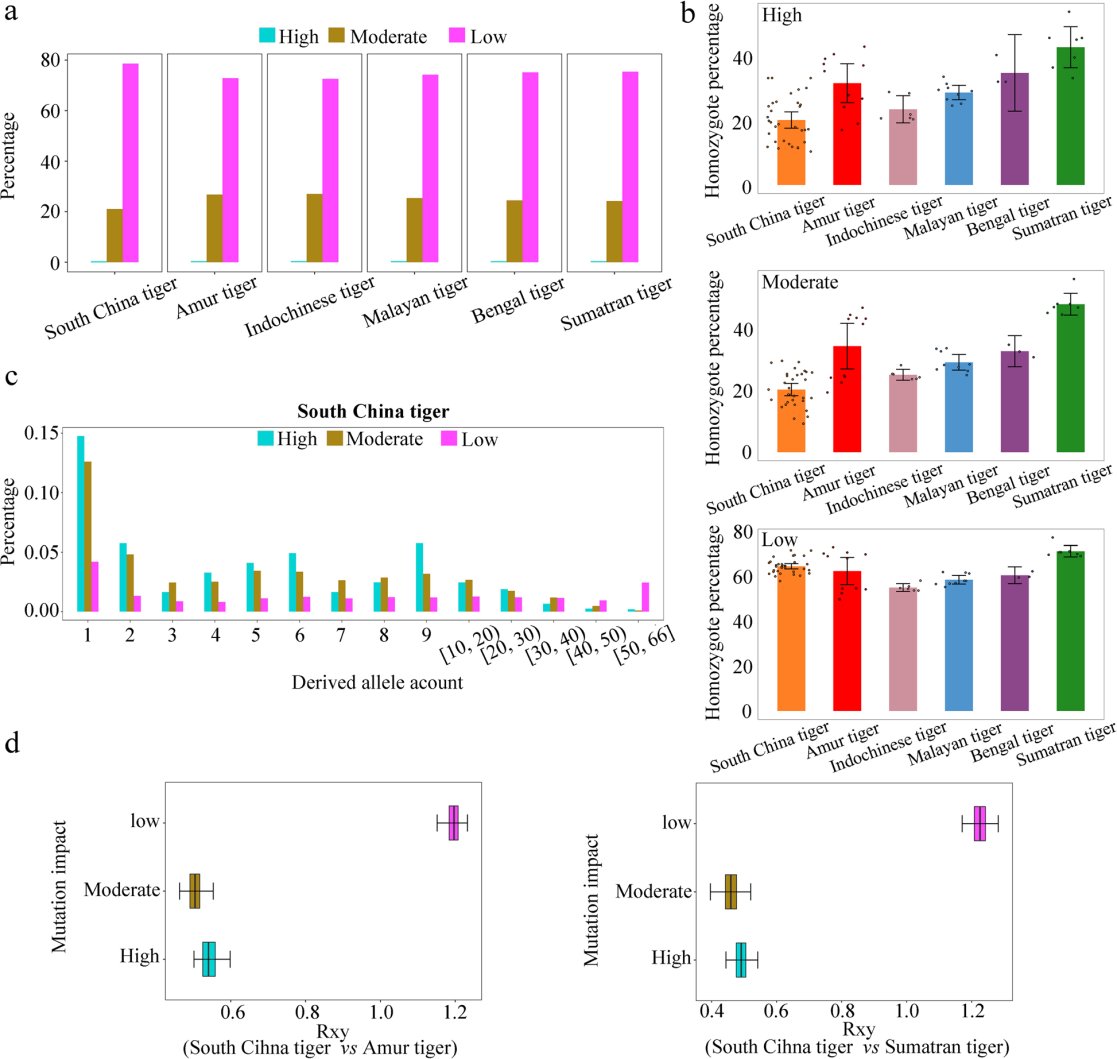
Comparison of deleterious mutations among different tiger subspecies. **a** Percentage of three categories of nearly neutral (low), mildly (moderate), and highly deleterious (high impact) mutations in different tiger subspecies. **b** Individual homozygote percentage per mutation category of different tiger subspecies. **c** Site frequency spectra for SNPs per mutation category in the South China tiger. Derived allele counts ≥ 10 are displayed as mean counts per interval. **d ***Rxy* analysis compares the South China tiger with the Sumatran and Amur tigers per mutation category. *Rxy* < 1 indicates a relative frequency deficit of the corresponding category

Because of the importance of managing deleterious mutations in conserved genomic elements for species conservation [49], we computed the proportion of deleterious mutations retained in potential coding regions of six tiger subspecies and obtained a total of 70,273 SNPs in three categories of high-, moderate-, and low-impact (nearly neutral) mutations, of which only 0.51% were highly deleterious as most of them were stop-gain mutations across six tiger subspecies (Additional file 1: Table S22). All tiger subspecies shared a similar distribution pattern in the proportions of these three categories of mutations (Fig. 4a and Additional file 1: Table S22). Nevertheless, the average number of homozygous sites with high- and moderate-impact mutations of the South China tiger (9.27 and 632.51, respectively) was significantly lower than other tiger subspecies (19.17–29.66 and 1255.67–2198.42, respectively) (*t* test, *P* < 0.01), while the average number of homozygous sites with low-impact mutations of the South China tiger (13131.88) was close to other tiger subspecies (11127.83–12914.29) (Additional file 1: Table S23), an indication of a stronger genetic purging of the high- and moderate-impact mutations from the South China tiger. Additionally, the average proportions of high- and moderate-impact mutations in a homozygous state were the lowest (20.24% and 20.47%, respectively) among the South China tiger compared with other tiger subspecies (23.59–42.94% and 25.29–48.34% for high- and moderate-impact mutations, respectively) (Fig. 4b and Additional file 1: Table S23), indicating a relatively effective genetic purging of homozygous genotypes of such mutations. Moreover, this was particularly true that the average proportion of homozygous genotypes of both high- and moderate-impact mutations to all deleterious mutations was much lower than that of the low-impact mutations in the South China tiger, a pattern largely shared by other tiger subspecies (Additional file 1: Table. S23 and Additional file 2: Figure S23). The distribution of derived alleles of such high- and moderate-impact mutations displayed a downward shift compared to the low-impact mutations in the South China tiger (Fig. 4c). Besides, we calculated *Rxy* [50] to estimate whether there was an excess or deficit of deleterious mutations in the South China tiger compared with the other tiger subspecies. The relative mutation loads of *Rxy* were reduced below 1 in both high- and moderate-impact mutations in the South China tiger compared with both Amur and Sumatran tigers (Fig. 4d). Altogether, these results indicated a relatively effective genetic purging of both high- and moderate-impact mutations in a homozygous state from the South China tiger following its population contraction with a controlled increase in inbreeding based on pedigree records, a phenomenon that was observed in other species [51–54].

## Discussion

To facilitate the best practice of conservation genomics based on high-density markers across the entire genomes [55] to mitigate potential inbreeding loads associated with deleterious mutations of all endangered tigers, we first assembled the best South China tiger genome and applied it as a reference to the analyses of all tiger genomic data [13, 20–23]. We analyzed the largest population of the South China tiger combined with other tiger subspecies and found that all six tiger subspecies phylogenetically differentiated from each other, despite gene flow between some tiger subspecies [1–3]. The admixture from the Amur, Indochinese, and Bengal tigers was found in the captive South China tiger population by previous genomic studies [1, 15], so the South China tiger was thought to be the most taxonomically controversial among all the tiger subspecies. From 2004, the Tiger TAG collected blood samples from all newborn tigers for genetic analysis to remove apparently admixed tigers from breeding, including the ptam_1 from the Chongqing Zoo [1] that was obviously a hybrid from the Indochinese tiger (Additional file 2: Figures S16 and S20). In this study, we found that the South China tiger had very limited genetic admixture from other tiger subspecies, validating very little or negligible genetic contamination in the captive South China tiger population. However, we only re-sequenced the whole genomes of 29 South China tigers collected from the four major zoos in China. As such, we shall continue and focus our efforts on collecting and re-sequencing additional whole genomes of most, if not all, reproductive South China tigers, to further fine-map and effectively manage their viable genomic landscapes along with unique genetic variations and putatively deleterious mutations.

Evaluation of genomic variations in all tiger subspecies showed that the Sumatran tiger had the lowest genetic diversity, while the South China tiger harbored moderate genetic variations. We also found genomic inbreeding in the Sumatran, Amur, and South China tigers. Moreover, the accumulation of large ROH (> 1 Mb) since the beginning of captive breeding has been resulting in relatively high genomic inbreeding and genetic burden in the South China tiger [56]. It was reported that the captive South China tiger population suffered from inbreeding depression in terms of high juvenile mortality and impaired adult fertility [12]. To control the inbreeding based on pedigree records, the captive South China tigers were permitted to mate only if their mate suitability indexes (MSI, ranging from 1 to 6 across all tigers) calculated by PMx software [57], were below 4. The MSI is a composite score that integrates four genetic components into a single index, including Delta GD, differences in Mk values, inbreeding coefficient, and unknown ancestry [58]. Therefore, the breeding plan based on the MSI may have assisted most deleterious mutations to be inherited as recessive alleles masked in heterozygous states and thus free of purifying selection [59, 60]. Our genomic analysis, however, revealed an efficient genetic purging of both high- and moderate-impact deleterious mutations from the South China tiger genomes. It is known that the purging reduces the frequency of deleterious mutations, depending on the degree of dominance and the magnitude of the deleterious effects. Because most high-impact mutations were stop-gain variants leading to loss of gene functions, the high-impact mutations were exposed to the highest strength of purging [51]. It was true that the average number of homozygous sites with high-impact mutations per individual (9.27) was much lower than those of the moderate- (632.51) and low-impact (13,131.88) mutations in the South China tiger genomes. Surprisingly, the lowest proportion of homozygous genotypes and relatively low *Rxy* estimates (< 1) were associated with both high- and moderate-impact mutations in the South China tiger genomes. This verified that both high- and moderate-impact mutations were more likely to be inherited as recessive alleles than low-impact mutations in the captive South China tiger population [12]. Based on recent founder effect and ongoing inbreeding, these deleterious mutations are expected to be continuously accumulated in the South China tiger genomes, therefore their potential impacts on fitness should be evaluated across generations of the captive South China tiger population [10, 11].

It is certain that all captive South China tigers are the descendants of two males and four female tigers [9]. The Shanghai and Guiyang founder lineages were formed during the 1970s, but the two lineages began to merge for breeding to minimize potential inbreeding depression since the mid-1980s [11]. The two genetic lineages based on current genome-wide SNPs and early mtDNA and microsatellite analyses [15] clearly mirrored the two founder lineages, due probably to historical allelic segregation and/or genetic drift among limited founders of separate geographical origins and relatively independent reproduction of the South China tigers in isolated zoos, as what was observed in the killer whale ecotypes [46] and highly inbred pigs [61]. The Tiger TAG has been in charge of the breeding and transfer plan for South China tiger, following a principle of priority to allow the tigers to breed in their original facilities first, which may have limited the exchange of breeding tigers among the zoos [11]. According to the studbook, the descendants of the Guiyang lineage were much fewer than those of the Shanghai lineage [9]. We also recognized that the tigers of the two lineages were not evenly distributed among the zoos, for example, the seven tigers sampled from the Guangzhou Zoo for this study all belonged to the Shanghai lineage. This calls for a genomics-assisted exchange plan of breeding tigers across all major zoos. In fact, the Shanghai and Guiyang lineages showed different reproductive performance and fitness among the newborn tigers [62]. Our genomic analysis detected the lowest genomic inbreeding of the South China tigers in the Shanghai Zoo. We therefore recommend a genomics-informed exchange of breeding tigers between these two founder/genomic lineages, so that to maximize the benefit of maintaining existing unique and critical genetic variations that are expected to outweigh the cost of increased genetic load within the captive South China tiger population [20].

## Conclusions

In this study, we de novo assembled the high-quality chromosome-level reference genome of a South China tiger and re-sequenced the whole genomes of 29 South China tigers collected from four major breeding zoos in China. The results indicated that the captive South China tigers included in our study (expect the pam_1) had limited genetic admixture from other tiger subspecies. The genetic diversity was slightly higher in the South China tiger than in the Sumatran tiger, while the South China tiger had high *F*_ROH_ values under longer ROH (> 1 Mb), indicating its recent inbreeding/founder events and/or population bottleneck/isolation. Although most high- and moderate-impact deleterious mutations in the South China tiger genomes may be masked as recessive alleles for their inheritance in heterozygous states, such deleterious SNPs have been effectively purged, when they were in homozygous states, from the South China tiger population. We recommend that individuals of the two genomic lineages could be merged to breed for minimizing a further loss of the unique and critical genetic variations. All the new findings from our present study demonstrated the power and effectiveness of concerted efforts to conserve the captive South China tiger in the past and shed light into a potentially bright future of these critically endangered cats. The captive South China tigers are the last hope of the tiger subspecies, we wish that they would have a success of reintroduction program in the future and to be another case as what is achieved in the protection of the Giant Panda, which was downlisted to 'vulnerable' on the IUCN Red List.

## Methods

### De novo genome sequencing

Blood samples of two male (Tuantuan and Kangkang) and ione female (Huanhuan) South China tiger were acquired from the Guangzhou Zoo for genome sequencing. All the DNA was extracted from blood using phenol-chloroform method [63]. All libraries were constructed at BGI (Shenzhen, China). For PacBio sequencing, genomic DNA from the Tuantuan was used to construct three libraries of 20-kb insert size using the SMRTbell Template Prep Kit 1.0 (Pacific Biosciences, USA), and the fragment size was selected using Blue Pippin (Labgene Scientific SA, Switzerland). Then, the library sequencing was performed on a PacBio RS II sequencer. For short reads sequencing, three short insert libraries (270, 500, and 800 bp) were constructed for Tuantuan and four long mate-paired insert libraries (2, 5, 10, and 20 kb) were constructed for Huanhuan. The libraries were constructed as per the manufacturer’s instructions (MGIEasy universal DNA Library Preparation Kit, BGI, China). All libraries were sequenced on the Illumina HiSeq X Ten Platform using 150-bp paired-end reads according to the Illumina protocols, except for the 800-bp library, which was sequenced on the HiSeq 2500 System using 125-bp paired-end reads.

### Estimation of genome size

We applied K-mer (17-mer) distribution analysis to estimate the South China tiger genome size using clean reads from the short-insert libraries. Genome size was calculated using the formula: Genome size = K-mer_number / K_depth of peak.

### Genome assembly

The PacBio data were de novo assembled using WTDBG-1.2.8 (https://github.com/ruanjue/wtdbg-1.2.8) and genomic contigs were polished with the Arrow program (https://www.pacb.com/support/software-downloads/) by aligning SMRT reads, which yielded an assembly (2.43 Gb in length) with N50 size at 6.24 Mb. We also aligned the Illumina X Ten data to the assembly using BWA (v0.7.15) [64] for error correction by Pilon (v1.22) [65] with the parameter “--mindepth 6”, which is an integrated tool for comprehensive variant detection and genome assembly improvement. The final assembly generated a total length of 2.42 Gb and N50 length at 6.20 Mb.

### Construction of optical genome maps

DNA of sufficient quality was extracted and labeled from the Tuantuan blood cells according to standard BioNano protocols (BioNano Genomics), after which nicking, labeling, repairing, and staining processes were implemented. We digested DNA using a specific single-stranded nicking endonuclease (Nt.BspQI). BioNano Solve (v3.0.1) [66] was used to produce optical maps with single molecules above 100 kb in size and six labels per molecule. The scaffold-level assembly had a N50 length at 31.62 Mb.

### Hi-C analysis

The Hi-C library was constructed from the blood cells of Kangkang according to the standard procedures of BGI and sequenced using the MGISEQ-2000 Platform. The Bionano-based scaffolds were anchored into a chromosome-scale assembly using a Hi-C proximity-based assembly approach. We aligned the Hi-C reads to scaffolds using bowtie2 (v2.2.5) [67] and interaction maps were generated following HiC-Pro (v2.5.0) [68] pipelines. The uniquely mapped read pairs were used as input for Juicer [69] and 3d-DNA [70] Hi-C analysis and scaffolding pipelines. The resulting Hi-C contact maps were visualized using Juicebox [71], and mis-assemblies and mis-joins were manually corrected based on neighboring interactions. The preliminary chromosome assembly was then generated and named as Amotig1.0. The Amotig1.0 genome was assessed by calculating the number of Hi-C read pairs in any two bins of 500 kb. The synteny analysis between the Amotig1.0 and the domestic cat genomes (Ensembl release 98, accessed in September 2019) was performed using the Mummer-4.0.0 software (https://github.com/gmarcais/mummer). The chromosome IDs in the Amotig1.0 genome were determined according to the synteny relationship with the cat genome. To evaluate the quality of the Amotig1.0 genome, we performed the BUSCO pipeline (version 5.0) [27] with orthologs database of mammalia_odb10.

### Genome annotation

We identified repetitive sequences in the Amotig1.0 genome using a combination of homology- and de novo-based methods. First, RepeatMasker [72] and RepeatProteinMask [72] were used to search repeat sequences against with Repbase database [73, 74]. Second, LTRharvest (GenomeTools v1.5.9) [75] and RepeatModeler (http://www.repeatmasker.org/RepeatModeler/) were used to build a de novo repeat library, and then repeats were annotated using RepeatMasker [72] with default parameters. Last, tandem repeats were detected using Tandem Repeats Finder (TRF) [76].

For gene structure prediction, we used homology-based prediction based on the protein sequences from five species (*Felis catus*, *Homo sapiens*, *Mus musculus*, *Panthera pardus*, and *Panthera tigris altaica*) downloaded from the Ensembl database (release 93). These protein sequences were mapped to the Amotig1.0 genome using TBLASTN (*E*-value cutoff: 1e^−5^) [77]. High-scoring segment pairs (HSPs) were concatenated using Solar (v0.9.6) [25]. GeneWise (v2.4.1) [78] was used to define accurate gene models. We then merged and filtered redundancy from different homology results based on the GeneWise score (≥ 0.4). To obtain the final gene set, transposons and single-exon genes without functional annotations were filtered out.

Gene functional annotations were assigned using BLASTP (BLAST+ v2.2.26) [77] against public databases, including the Swiss-Prot (release-2017_09) [79], TrEMBL (release-2017_09) [79], KEGG (v84.0) [80], COG [81], and NCBI nucleotide collection nr/nt (https://www.ncbi.nlm.nih.gov/nucleotide/, v20170924). The motifs and domains in the protein sequences were annotated using InterProScan (v5.16-55.0) [82].

The tRNA genes were predicted using tRNAscan-SE (v1.3.1) [83] with eukaryote parameters. The rRNA fragments were identified by aligning the rRNA template sequences from the Human Rfam database [84] using BLASTN (BLAST+ v2.2.26) [77] (*E*-value 1e^−5^). The snRNAs and miRNAs were searched via a two-step method: i.e., aligned with BLAST and then searched with INFERNAL (infernal-1.1.1) [85] against the Rfam database.

### Sampling information and whole genome re-sequencing

We collected a total of 30 specimens, including 29 South China tigers (*P. t. amoyensis*) from four major zoos in China and a domestic cat (*Felis catus*) from the Guangzhou Zoo, China (Supplementary Table S11). The 29 South China tigers were born in 1999 to 2018, and the blood samples of the individuals were collected in four major city zoos in 2018 and 2019. Genomic DNA was extracted from whole blood using the DNeasy Blood & Tissue Kit (QIAGEN, Valencia, California, USA) following the manufacturer’s protocols. For each sample, one 350 bp size of DNA library was constructed according to the manufacturer’s protocols (Illumina). The constructed libraries were sequenced using the Illumina Hiseq X Ten platform for 150 bp paired-end reads.

### Quality control

To ensure reads were reliable and without artificial bias (e.g., low-quality paired reads that result from base-calling duplicates and adapter contamination), we conducted a series of quality control (QC) procedures, as follows:

1. Removed reads with ≥ 1% unidentified nucleotides (*N*).
2. Removed reads with > 40% bases having phred quality < 20.
3. Removed reads with > 10 nucleotides overlapping the adapter (allowing ≤ 10% mismatches).

### Read alignment and variant calling

We used BWA [86] to align the clean reads of each sample against our newly assembled South China tiger genome (settings: mem -t 4 -k 32 -M -R). Alignment files were converted to BAM files using SAMtools (settings: -bS -t) (v-0.1.19) [87]. In addition, potential PCR duplications were removed using Picard (http://broadinstitute.github.io/picard/). We called SNPs using the HaplotypeCaller approach implemented in the Genome Analysis Toolkit (GATK) package [88]. Filtering criteria were as follows:

1. SNPs with QD < 2.0; FS > 60.0; MQ < 40.0; QUAL < 30; DP < 4.0; MQRankSum < -12.5; and ReadPosRankSum < -8.0 were filtered.
2. Multi-nucleotide polymorphisms were ignored.
3. SNPs within 5 bp of a gap were filtered.
4. Overall depth (for all individuals) was > 1/3× and < 3×.
5. Unobserved variant allele constituted < 10%.

A total of 54,067,600 high-quality SNPs were retained for subsequent analyses after filtering. Gene-based SNP annotation was performed using ANNOVAR [89].

### Phylogenetic and population genetic analysis

We selected genome-wide 54,067,600 SNPs for phylogenetic construction with a cat as outgroup and 10,205,707 SNPs for PCA and population structure analyses components. PCA was performed using PLINK (v1.9) (settings: --bfile --pca –noweb) [90]. Genetic structure was inferred using ADMIXTURE (v1.3) [91], with implementation of a block-relaxation algorithm (settings: --cv -m=block). To explore convergence of individuals, we predefined the number of genetic clusters *K* from 2 to 9. We calculated the *p*-distance matrix using VCF2Dis (https://github.com/BGI-shenzhen/VCF2Dis) and a NJ tree was generated using the R package APE and 100 bootstraps were run for a reliable tree [92].

### Calculation of genetic diversity

Pairwise nucleotide diversity *θ*_π_ and Watterson’s estimator *θ*_w_ [93] within a tiger subspecies were calculated using a sliding-window approach (20-kb windows sliding in 10-kb steps). Genetic differentiation between tiger subspecies was calculated using the pairwise fixation index *F*_ST_ [94].

### Identity-by-state (IBS) and identity-by-descent (IBD) analyses

To evaluate the similarity between two tigers within a tiger subspecies, genome-wide IBS pairwise identities were calculated using the toolset SNPRelate [95] in the R package. Based on the matrix of genome-wide IBS pairwise distances, we performed multidimensional scaling and cluster analyses and determined the groups using a permutation score. We also calculated the pairwise IBD using PLINK (v1.9) (settings: --file --genome --min 0.05) [90].

### Introgression and demographic history analyses

We separated the South China tiger population into three classes (i.e., lineage 1, lineage 2, and ptam1) based on phylogeny. TreeMix (v1.13) [96] was used to detect gene flow between the tiger subspecies based on genome-wide allele frequency data at individual SNPs. We first constructed a maximum-likelihood tree for six tiger subspecies using blocks of 10,000 SNPs. The number of migration events was set from 1 to 6. We calculated introgression among six tiger subspecies using Patterson’s D-statistic (ABBA-BABA test) [97], with the cat as the outgroup, and tested the proportions of admixture events (*f4*-ratio) within each South China tiger using Dsuite (v0.4) [44], we calculated the mean introgression ratio between each South China tiger and other tiger subspecies.

We used the PSMC [98] model to reconstruct demographic history. To estimate effective ancestral population size changes for individual tiger subspecies, we selected individuals with high sequencing depth to ensure the quality of the consensus sequence. SNPs were detected using SAMtools [87], sites were filtered based on a minimum depth (DP = 4) and the highest depth (DP = 50) and mapping quality (Q = 20). We only retained autosomal SNPs. Parameters were set to: -N30, -t15, -r5, -p‘4+25*2+4+6’. A mutation rate of 3.5 × 10^−9^ per base per generation and generation time of 5 years was used.

### Analysis of runs of homozygosity

Regions of homozygosity were extracted for all chromosomes of all individuals based on SNP information. PLINK (v1.9) [90] was used to detect ROH via a sliding window approach, with the following parameters: ‘--homozyg-window-snp 100 --homozyg-window-het 2 --homozyg-window-missing 5 --homozyg-snp 100 --homozyg-kb 100 --homozyg-density 10 --homozyg-gap 100’.

### Inbreeding coefficient

We measured individual inbreeding using the genomic inbreeding coefficients *F*_H_ [48], which is the fraction of IBD of the two alleles in a diploid individual from a common ancestor. *F*_H_ was calculated using PLINK (v1.9) [90]. Alternatively, individual genomic inbreeding coefficients was also measured using *F*_ROH_ [99], which is an estimate of ROH proportion in an individual genome.

### Identification of deleterious mutations

The variants leading to functional changes were regarded as candidates of deleterious mutations. Thus, we only analyzed the SNPs in all potential coding regions, while the domestic cat allele was regarded as an ancestral allele. SnpEff (v4.3t) [100] was used for genetic variant annotation and functional effect prediction.

## Supplementary Information

**Additional file 1: Table S1.** Sample characteristics and genome sequencing information before and after filtering. **Table S2.** Sequencing data statistics. **Table S3.** 17-mer statistic information. **Table S4.** Genome assembly information. **Table S5.** Sizes of individual chromosomes. **Table S6.** Assessment of completeness of the South China tiger genome assembly. **Table S7.** Summary of repeat contents. **Table S8.** Statistics of gene structure prediction. **Table S9.** Result of gene functional annotation. **Table S10.** Summary of non-coding RNA in genome. **Table S11.** Sampling information included in the analyses. **Table S12.** Re-sequencing data statistics in this study. **Table S13.** Genome mapping and coverage information for all accessions analyzed in this study. **Table S14.** Number and distribution of SNPs in each genome. **Table S15.** Distribution of whole-genome SNPs in different subspecies/species. **Table S16.** Average genome-wide nucleotide diversity in six tiger subspecies. **Table S17.** Pairwise *F*_ST_ values between six tiger subspecies. **Table S18.** ABBA-BABA estimates. Only results with |Z score| > 3 were remained. **Table S19. ***f*4-ratio calculated between individual South China tigers and other tiger subspecies. Only results with FDR < 0.05 were retained. **Table S20.** Pairwise relatedness estimates based on allelic identity-by-descent (IBD). **Table S21.** Inbreeding coefficients and ROH information. **Table S22.** Deleterious mutations segregating across and within six tiger subspecies. **Table S23.** Individual homozygote and heterozygote SNP counts per impact category in each tiger subspecies.

**Additional file 2: Figure S1.** Distribution of 17-mer depth for estimating the genome size. The x-axis represents k-mer depths while y-axis represents the proportions. The blue line represents the proportion of 17-mer in each depth. The peak depth is at 25-fold and total number of 17-mer is 61,791,522,108. The South China tiger (*P. t. amoyensis*) genome size was estimated to be 2471.66 Mb from the formula: Genome size = K-mer_number/K_depth of peak. **Figure S2.** Hi-C chromosomal contact heat map. A 500 kb resolution was used to calculate the number of Hi-C read pairs in any two bins. **Figure S3.** The collinearity between the South China tiger and domestic cat (*Felis catus*) genomes. Each dot represents an aligned region while the minimum length is 10 kb. The red dot represents forward comparison and the blue dot reverse comparison. **Figure S4.** Q30 and GC content for each sample. The average Q30 and GC content are 93.24% and 42.04%, respectively. Their very low variation reflected our re-sequencing data to be high quality. **Figure S5.** The distribution of mapping rates and average mapping depths for each individual. The depths ranged from 12.91× to 18.96 × while the rates varied from 96.03% to 98.92%. **Figure S6.** The distribution of the SNP number and frequency. Number of genome-wide SNPs (top) and frequency of SNPs per 1 kb of each tiger sample (bottom). **Figure S7.** Nucleotide diversity π estimates of six tiger subspecies. **Figure S8.** Heterozygosity statistics of genome-wide SNPs. (a) Observed heterozygosity across all individual genomes of each tiger subspecies. (b) Genomic heterozygosity in each tiger subspecies at population level. **Figure S9.** Pairwise *F*_ST_ values between six tiger subspecies. The weighted *F*_ST_ values are shown above the diagonal while their standard deviations below the diagonal. **Figure S10.** Principal component analysis using genome-wide SNPs of six tiger subspecies. **Figure S11.** A maximum likelihood tree was built using TreeMix software with whole-genome sequencing data of six tiger subspecies and cat. **Figure S12.** Cluster analysis based on the matrix of genome-wide identity-by-state (IBS) pairwise distances between six tiger subspecies determined by a permutation score. **Figure S13.** Analysis of genome-wide average identity-by-state (IBS) pairwise identities between six tiger subspecies. **Figure S14.** Population genetic structure of the South China tigers estimated by the ADMIXTURE. **Figure S15.** Demographic histories of tiger subspecies. PSMC analysis shows the change in effective population size over time. The dash lines represent 100 bootstraps. The representative individuals sequenced at a high read coverage were selected for each graph, South China tiger (ptam_4, mean 25.38×), Amur tiger (ptal_1, mean 14.76×), Indochinese tiger (ptco_2, mean 13.98×), Malayan tiger (ptja_7, mean 13.13×), Bengal tiger (ptti_3, mean 13.79×), and Sumatran tiger (ptsu_1, mean 13.41×). **Figure S16.** The results of Dsuite. Heatmap showing statistical support for introgression between pairs of tiger subspecies. Cells in the heatmap indicate the pairwise Z score values between the branch b identified on the expanded tree on the Y axis (relative to its sister branch) and the taxa P3 identified on the X-axis. The grey color indicates the none. (a) Result of branch for South China tiger (lineage 1) with the other tiger subspecies populations. (b) Result of branch for South China tiger (lineage 2) with the other tiger subspecies populations. (c) Result of branch for South China tiger (ptam1 individual) with the other tiger subspecies populations. **Figure S17.** The graph of ABBA-BABA test. The values of D (pop1, pop2; pop3, pop4) >0 indicates that there are gene flows between pop1 and pop3. Here, only the introgression occurred in South China tiger will be shown. (a) D-statistic for South China tiger (lineage 1) with other tiger subspecies. (b) D-statistic for South China tiger (lineage 2) with other tiger subspecies. (c) D-statistic for South China tiger (ptam1 individual) with other tiger subspecies. **Figure S18.** Plot of inferred introgression between the South China tiger lineage 1 and other tiger subspecies populations detected by the TreeMix method. The scale bar shows 10 times the average standard error of the entries in the sample covariance matrix. **Figure S19.** Plot of inferred introgression between the South China tiger lineage 2 and other tiger subspecies populations detected by the TreeMix method. The scale bar shows 10 times the average standard error of the entries in the sample covariance matrix. **Figure S20.** Plot of inferred introgression between the South China tiger ptam_1 individual and other tiger subspecies populations detected by the TreeMix method. The scale bar shows 10 times the average standard error of the entries in the sample covariance matrix. **Figure S21.** Genomic inbreeding coefficients *F*_H_ in each tiger subspecies. **Figure S22.** The plot of three inbreeding coefficients (*F*_P_, *F*_H_, and *F*_ROH_) of the South China tiger. **Figure S23.** Comparison of homozygote and heterozygote percentage of per mutation category among six tiger subspecies. The significant value was calculated by *t*-test.

## Data Availability

Genome assemblies and DNA sequencing data were deposited in the National Genomics Data Center (NGDC, https://ngdc.cncb.ac.cn) under Project no. PRJCA006384. The de novo genome of the South China tiger was deposited under Accession ID GWHBEIN00000000 [28]. The re-sequencing genomic data of 29 South China tigers and a domestic cat were deposited under Accession ID CRA004909 [37].

## Abbreviations

SNPs: Single nucleotide polymorphisms
PCA: Principal component analysis
NJ: Neighbor-joining
IBD: Identity-by-descent
ROH: Runs of homozygosity
*F*_p_: Pedigree-based inbreeding coefficients
TRF: Tandem Repeats Finder
HSPs: High-scoring segment pairs
QC: Quality control
GATK: Genome Analysis Toolkit
IBS: Identity-by-state
