# Supplementary material for: Population genomic analysis provides evidence of the past success and future potential of South China tiger captive conservation
Source: BMC Biol. 2023 Apr 18;21:64. doi: 10.1186/s12915-023-01552-y (PMC10111772; doi:10.1186/s12915-023-01552-y)
Supplement: Supplementary file 2 — Additional file 2: Figure S1. Distribution of 17-mer depth for estimating the genome size. The x-axis represents k-mer depths while y-axis represents the proportions. The blue line represents the proportion of 17-mer in each depth. The peak depth is at 25-fold and total number of 17-mer is 61,791,522,108. The South China tiger (P. t. amoyensis) genome size was estimated to be 2471.66 Mb from the formula: Genome size = K-mer_number/K_depth of peak. Figure S2. Hi-C chromosomal contact heat map. A 500 kb resolution was used to calculate the number of Hi-C read pairs in any two bins. Figure S3. The collinearity between the South China tiger and domestic cat (Felis catus) genomes. Each dot represents an aligned region while the minimum length is 10 kb. The red dot represents forward comparison and the blue dot reverse comparison. Figure S4. Q30 and GC content for each sample. The average Q30 and GC content are 93.24% and 42.04%, respectively. Their very low variation reflected our re-sequencing data to be high quality. Figure S5. The distribution of mapping rates and average mapping depths for each individual. The depths ranged from 12.91× to 18.96 × while the rates varied from 96.03% to 98.92%. Figure S6. The distribution of the SNP number and frequency. Number of genome-wide SNPs (top) and frequency of SNPs per 1 kb of each tiger sample (bottom). Figure S7. Nucleotide diversity π estimates of six tiger subspecies. Figure S8. Heterozygosity statistics of genome-wide SNPs. (a) Observed heterozygosity across all individual genomes of each tiger subspecies. (b) Genomic heterozygosity in each tiger subspecies at population level. Figure S9. Pairwise FST values between six tiger subspecies. The weighted FST values are shown above the diagonal while their standard deviations below the diagonal. Figure S10. Principal component analysis using genome-wide SNPs of six tiger subspecies. Figure S11. A maximum likelihood tree was built using TreeMix software with whole-genome sequen [file 12915_2023_1552_MOESM2_ESM.doc]

**Population genomic analysis provides evidence of the past success and future potential of South China tiger captive conservation**

**Chen Wang et al.**

**Supplementary Information**

**Additional file 2.**

**
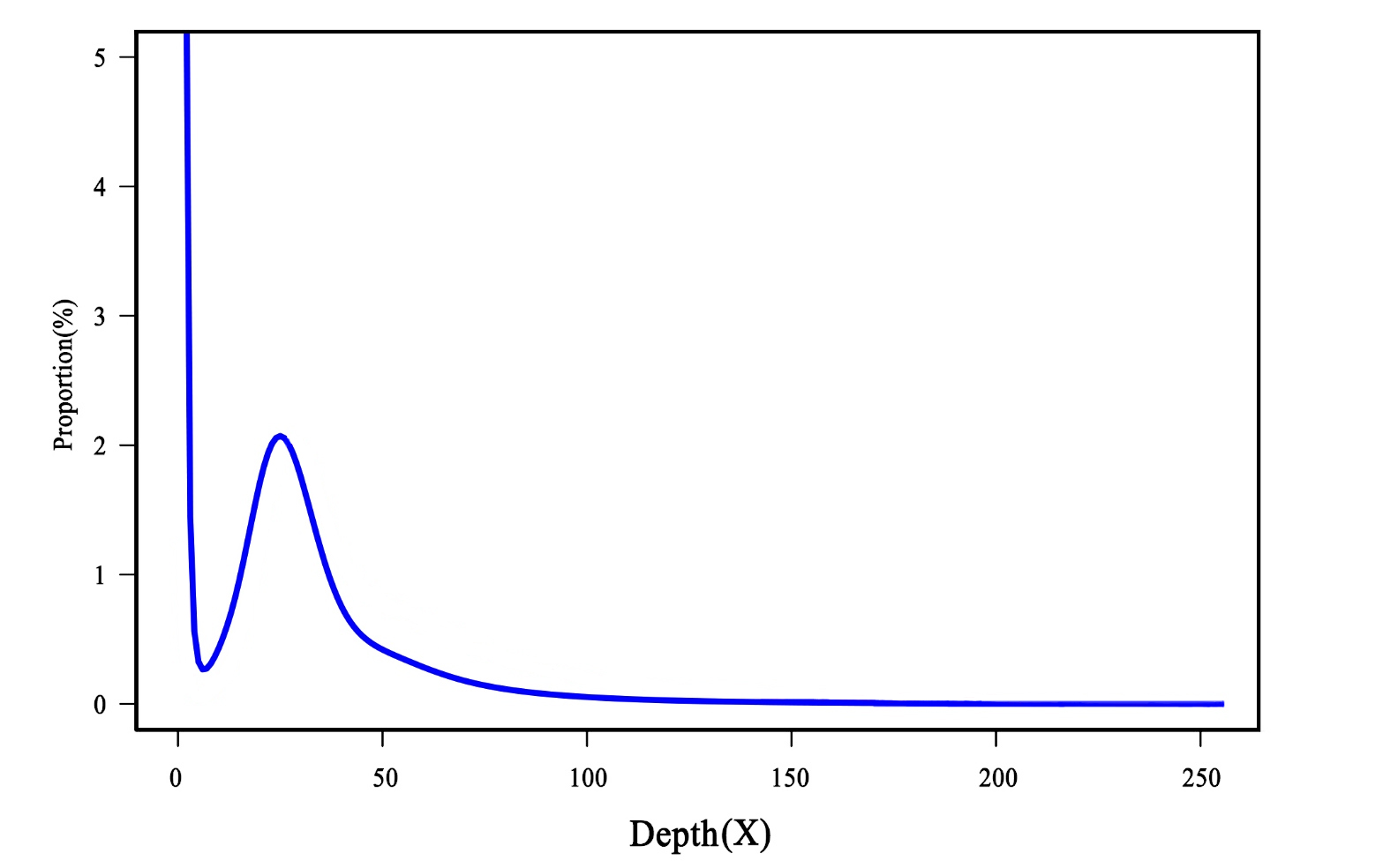
**

**Figure S1.** Distribution of 17-mer depth for estimating the genome size. The x-axis represents k-mer depth while y-axis represents the proportion. The blue line represents the proportion of 17-mer in each depth. The peak depth is at 25-fold and total number of 17-mer is 61,791,522,108. The South China tiger (*P. t. amoyensis*) genome size was estimated to be 2471.66 Mb from the formula: Genome size = K-mer_number/K_depth of peak.

**
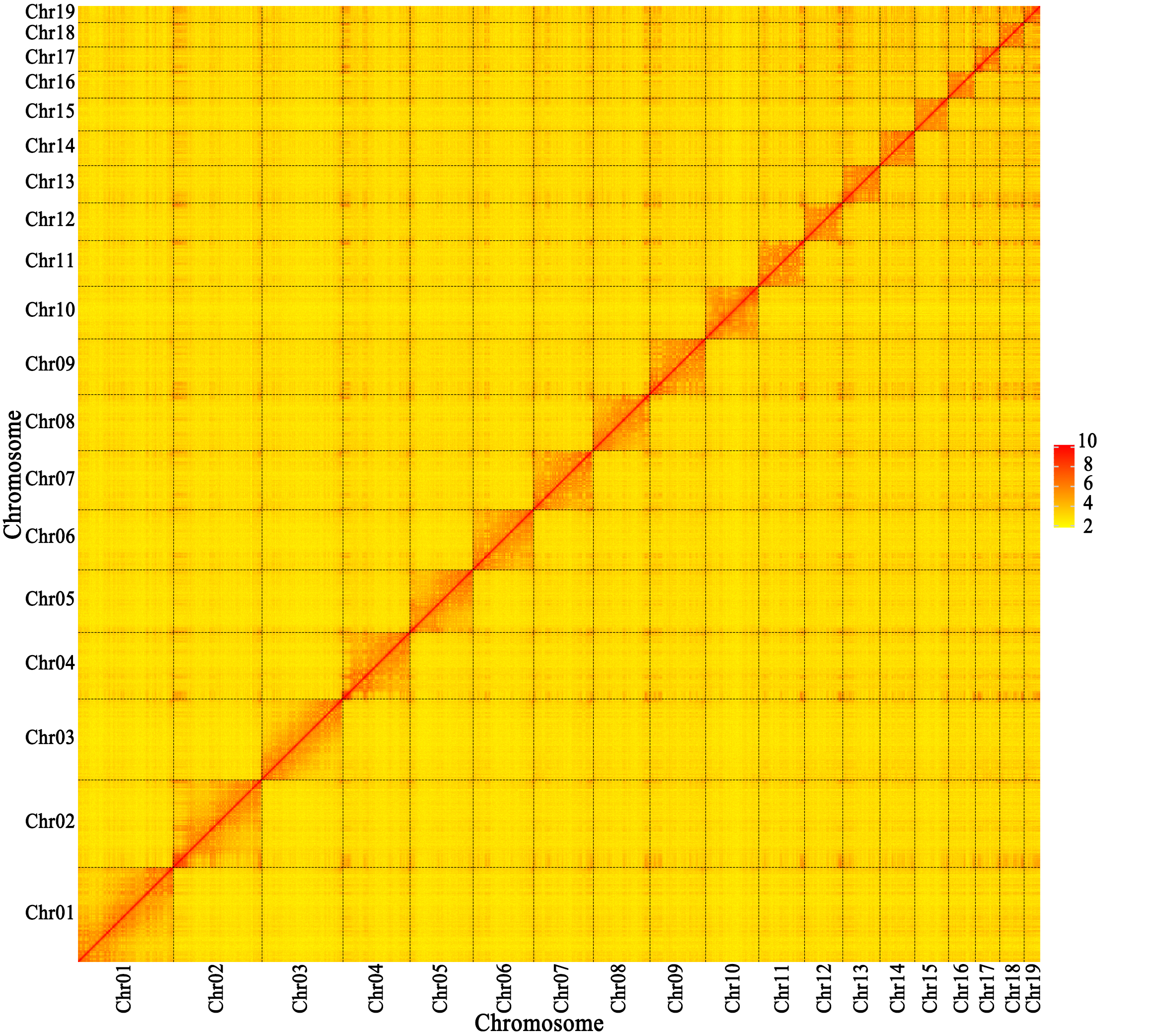
**

**Figure S2.** Hi-C chromosomal contact heat map. A 500 kb resolution was used to calculate the number of Hi-C read pairs in any two bins.

**
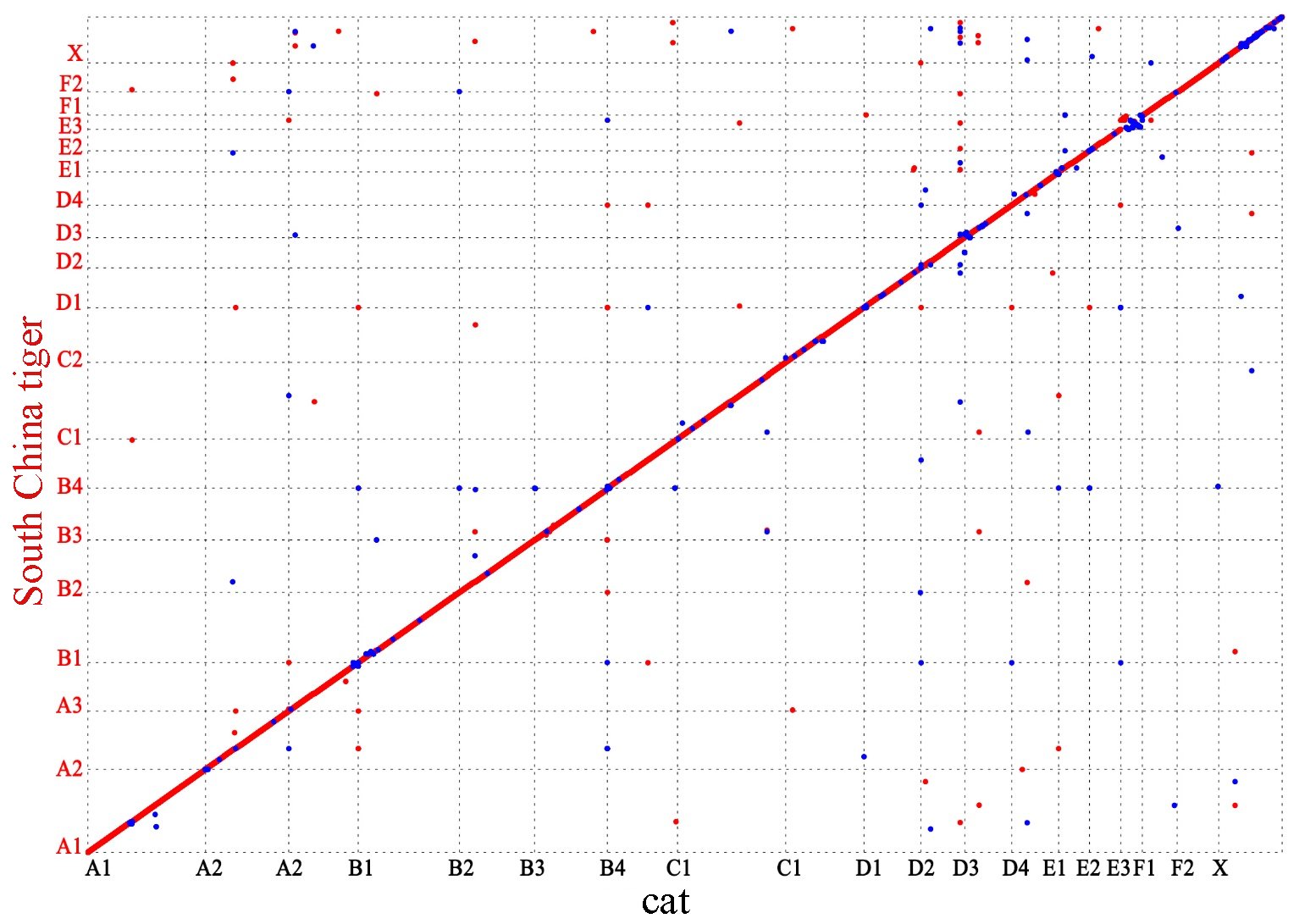
**

**Figure S3.** The collinearity between the South China tiger and domestic cat (*Felis catus*) genomes. Each dot represents an aligned region while the minimum length is 10 kb. The red dot represents forward comparison, and the blue dot represents reverse comparison.

**
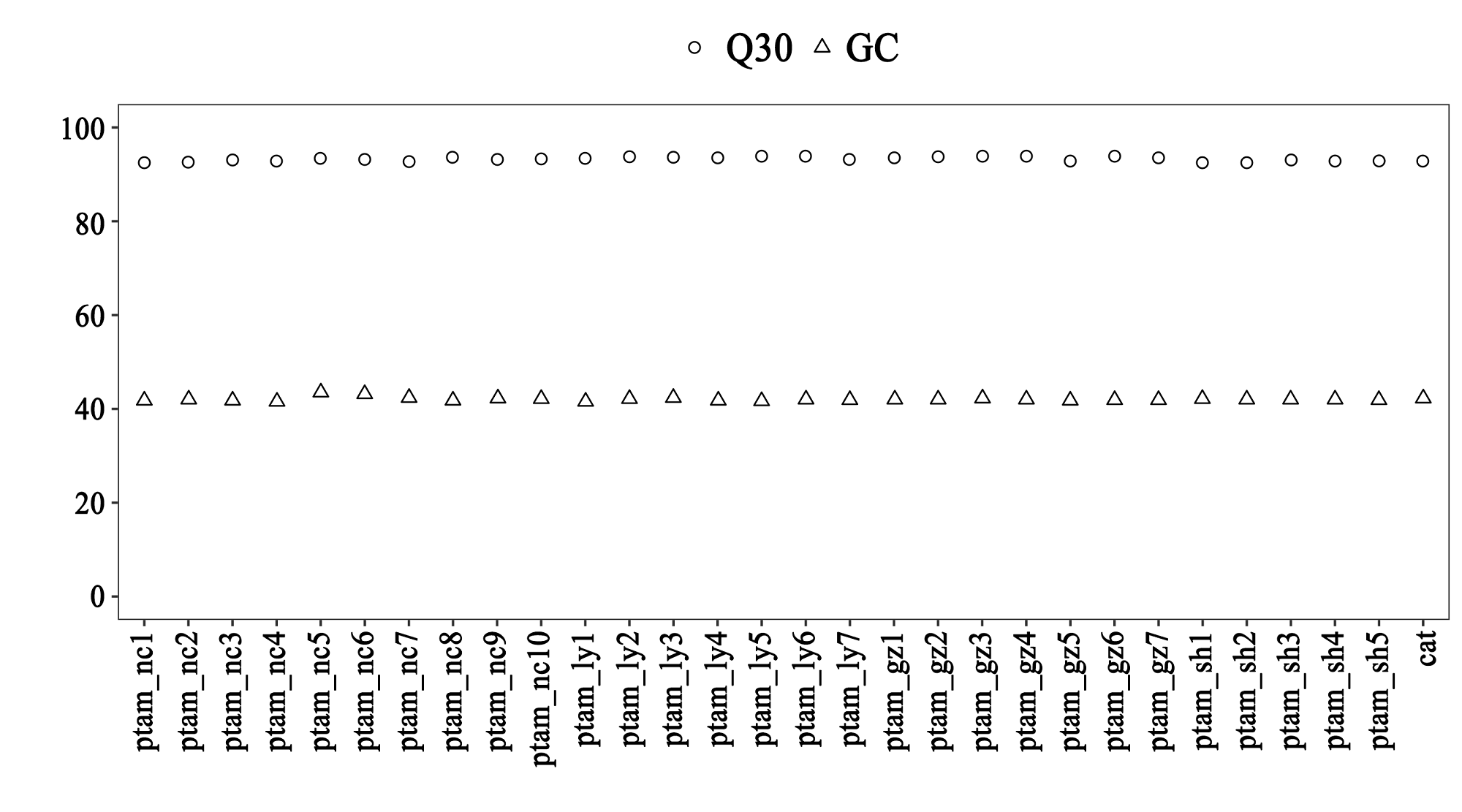
**

**Figure S4.** Q30 and GC content for each samples. The average Q30 and GC content are 93.24% and 42.04%, respectively. Their very low variation reflected our re-sequencing data to be high quality.

**
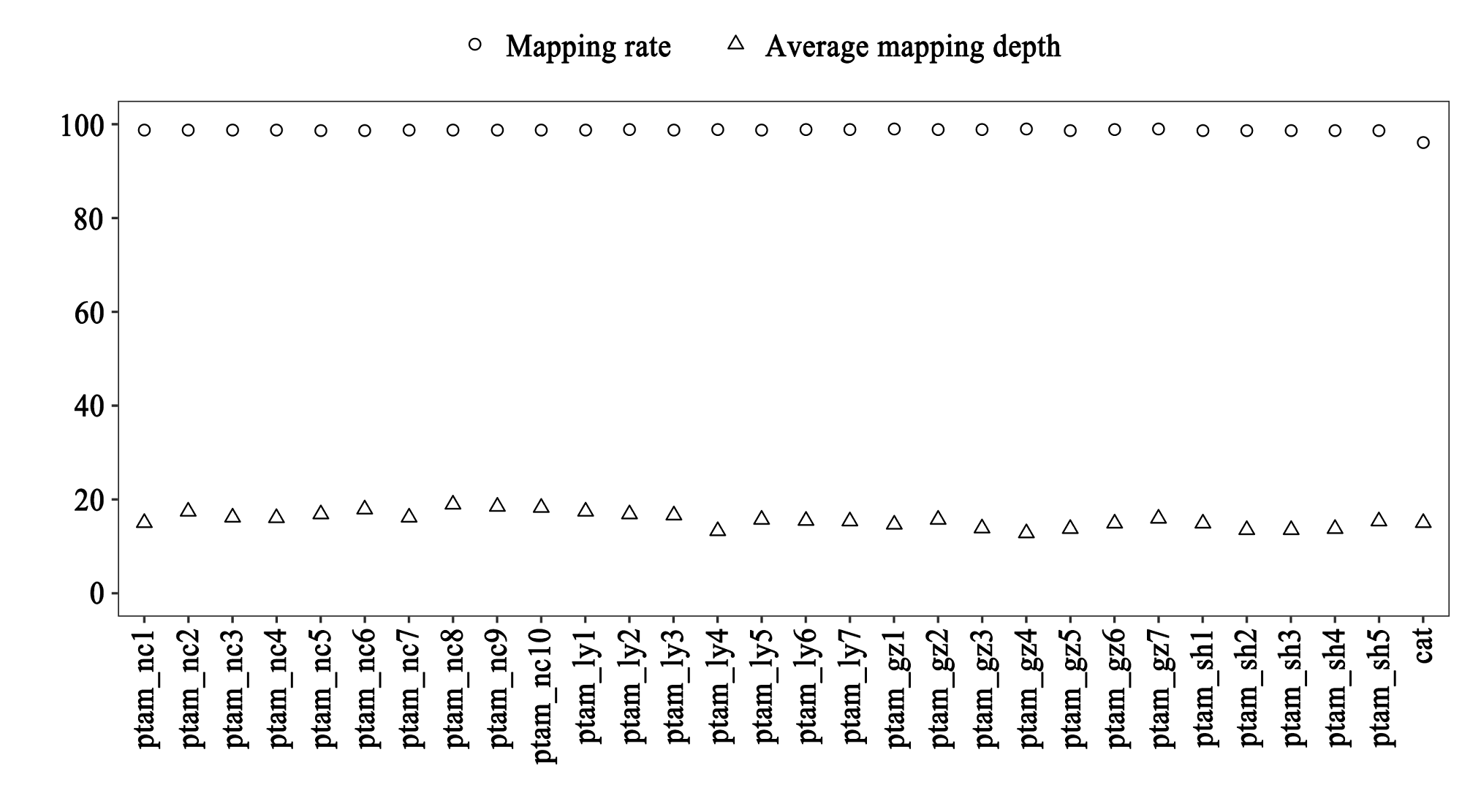
**

**Figure S5.** The distribution of mapping rates and average mapping depths for each individual. The depths ranged from 12.91× to 18.96 × while the rates varied from 96.03% to 98.92%.


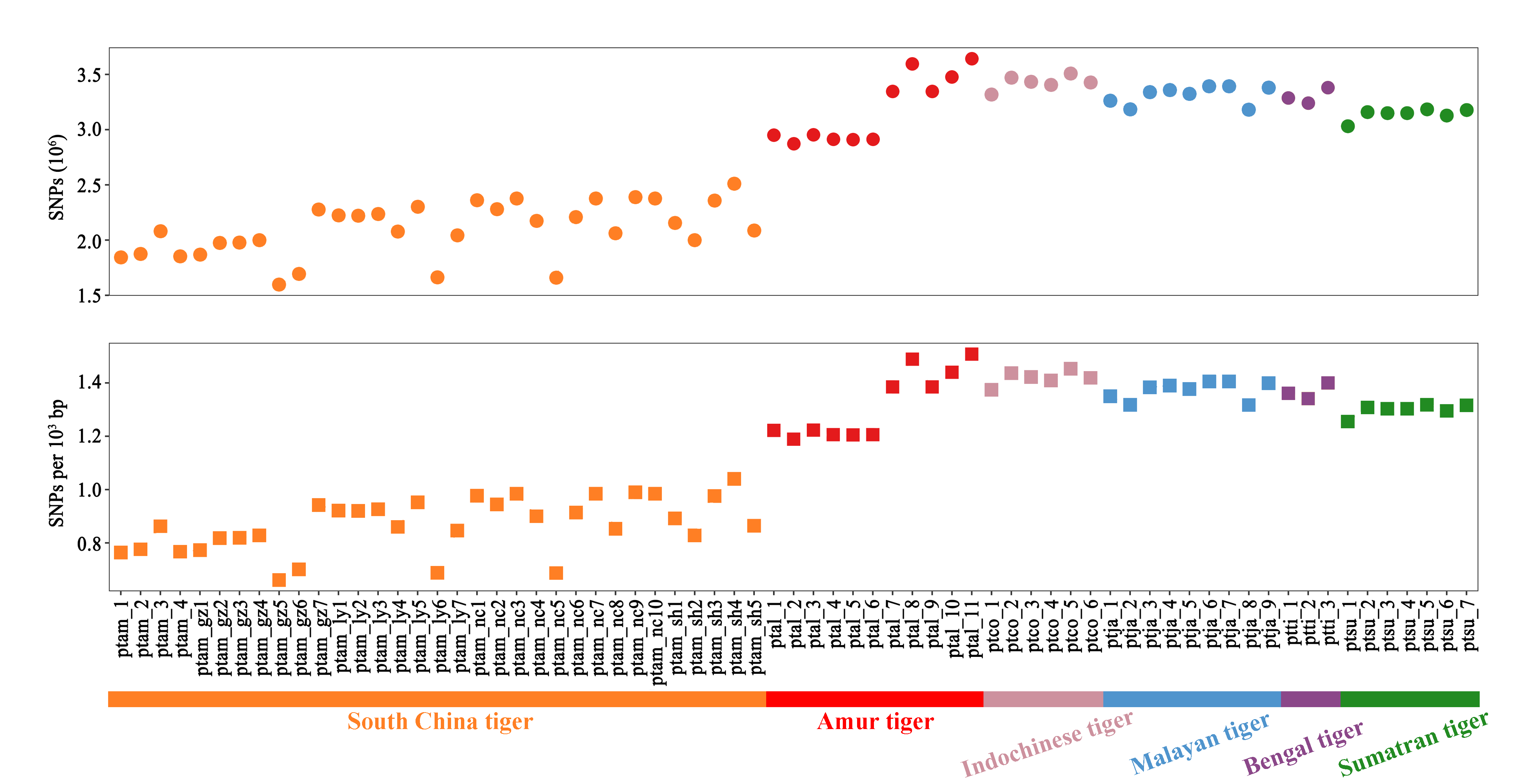


**Figure S6.** The distribution of the SNP number and frequency. Number of whole genome SNPs (top) and frequency of SNPs per 1 kb of each tiger sample (bottom).


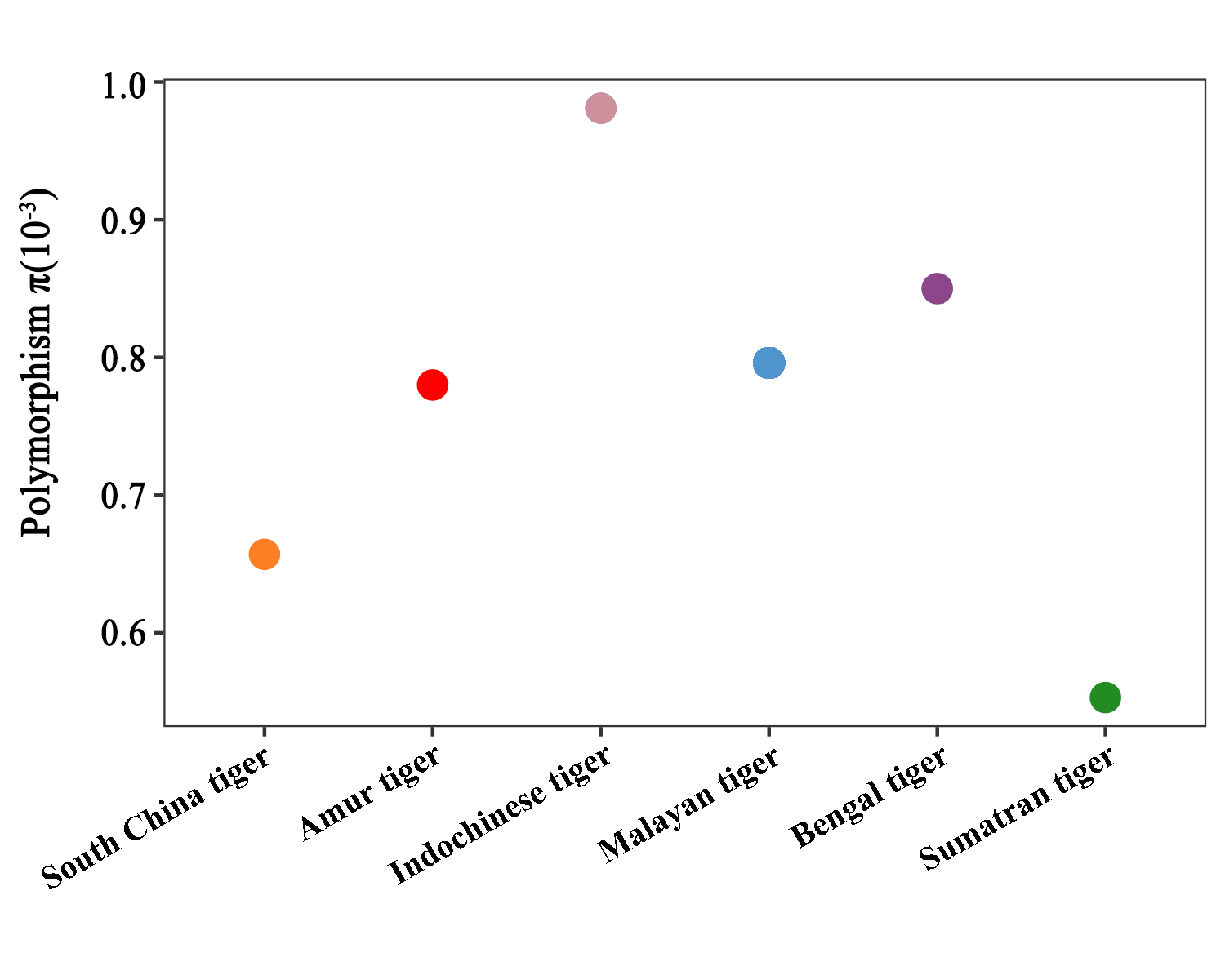


**Figure S7.** Nucleotide diversity π estimates of six tiger subspecies.


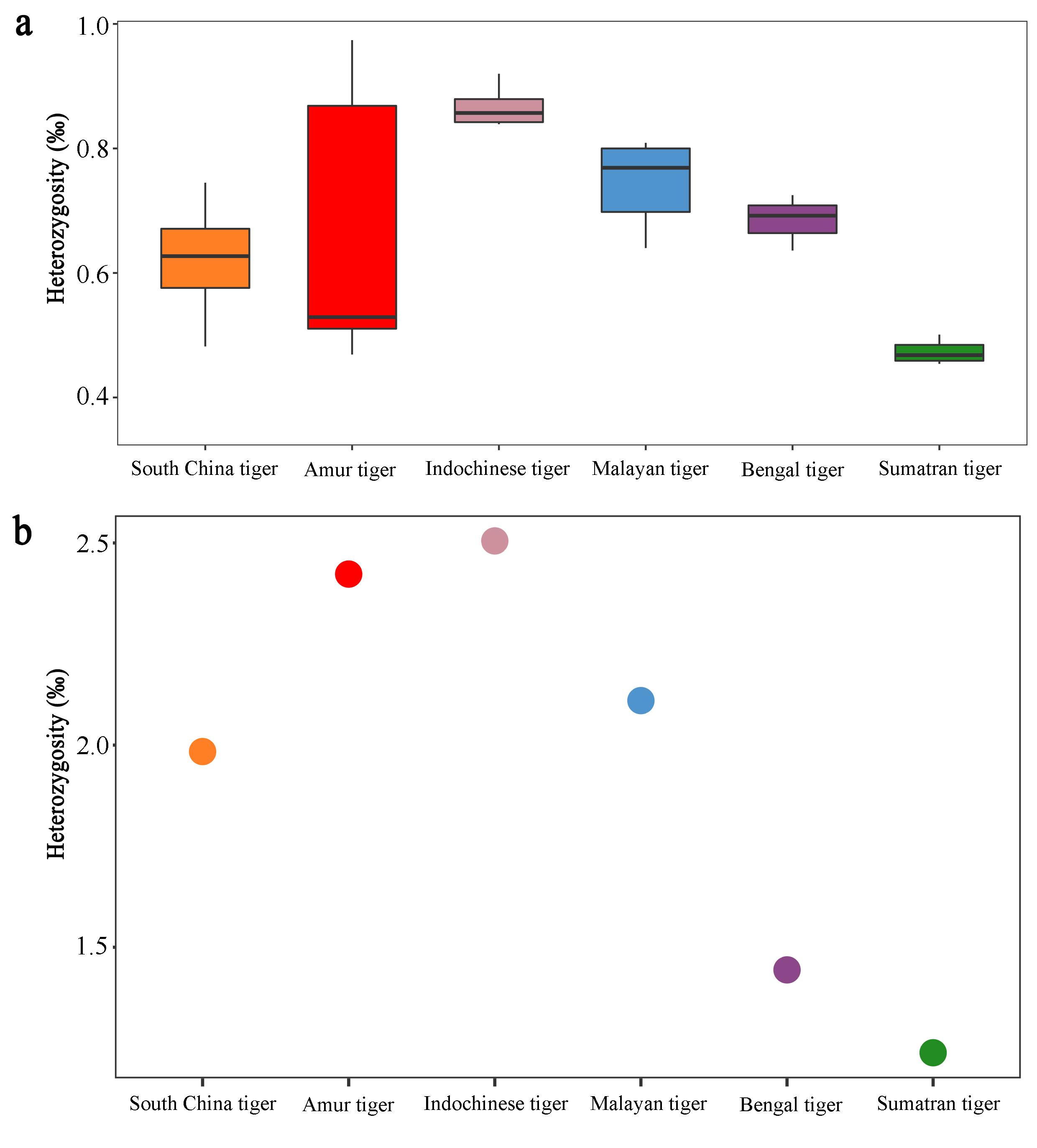


**Figure S8.** Heterozygosity statistics of genome-wide SNPs. (a) Observed heterozygosity across all individual genomes of each tiger subspecies. (b) Genomic heterozygosity in each tiger subspecies in population level.


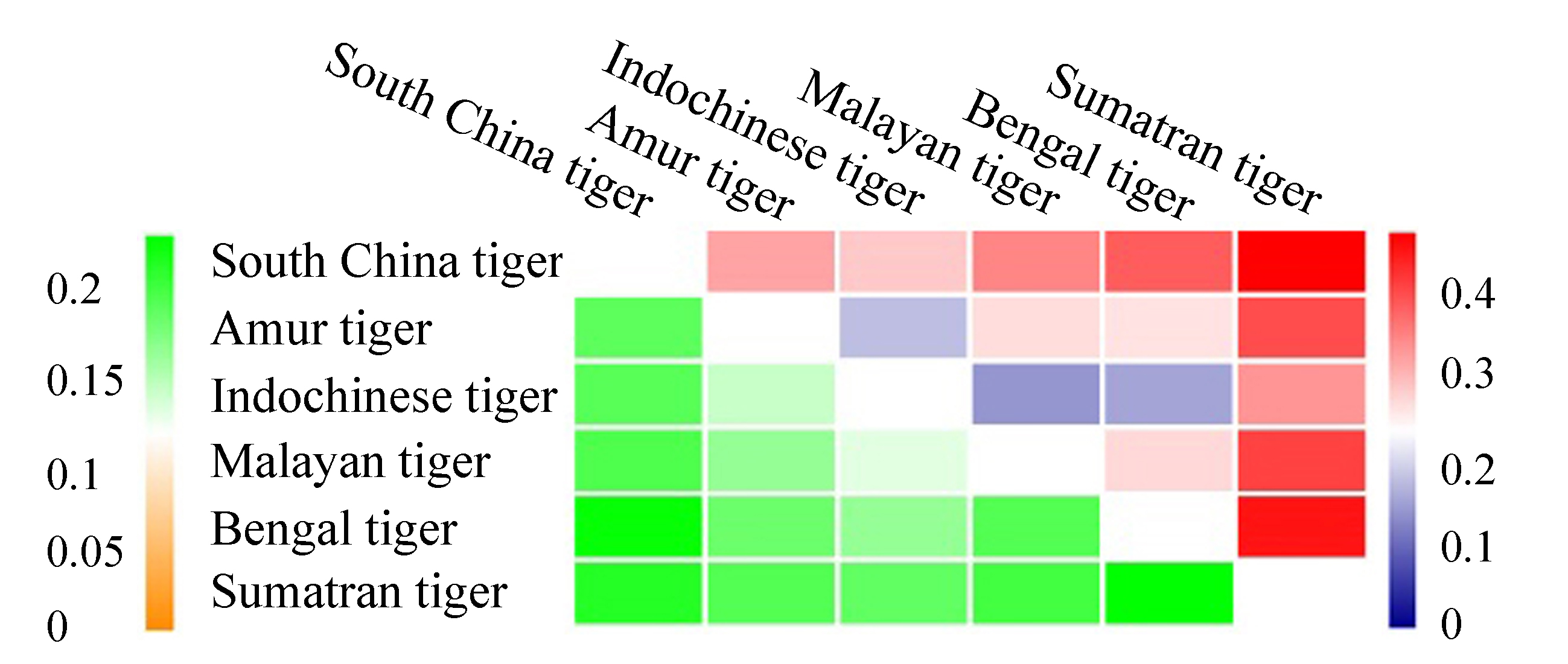


**Figure S9**. Pairwise *F*_ST_ values between six tiger subspecies. The weighted *F*_ST_ values are shown above the diagonal while their standard deviations below the diagonal.


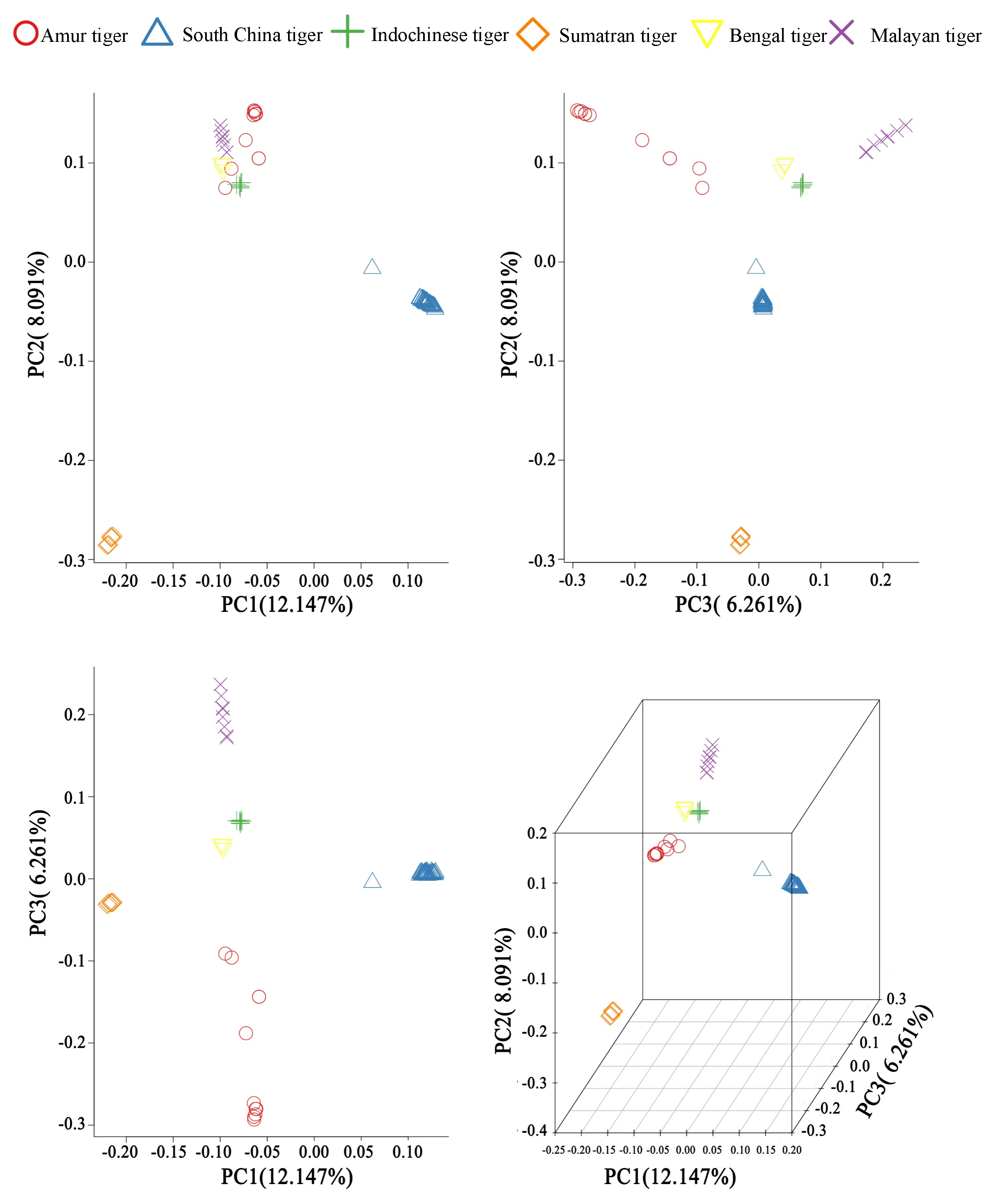


**Figure S10.** Principal component analysis using whole genome-wide SNPs of six tiger subspecies.


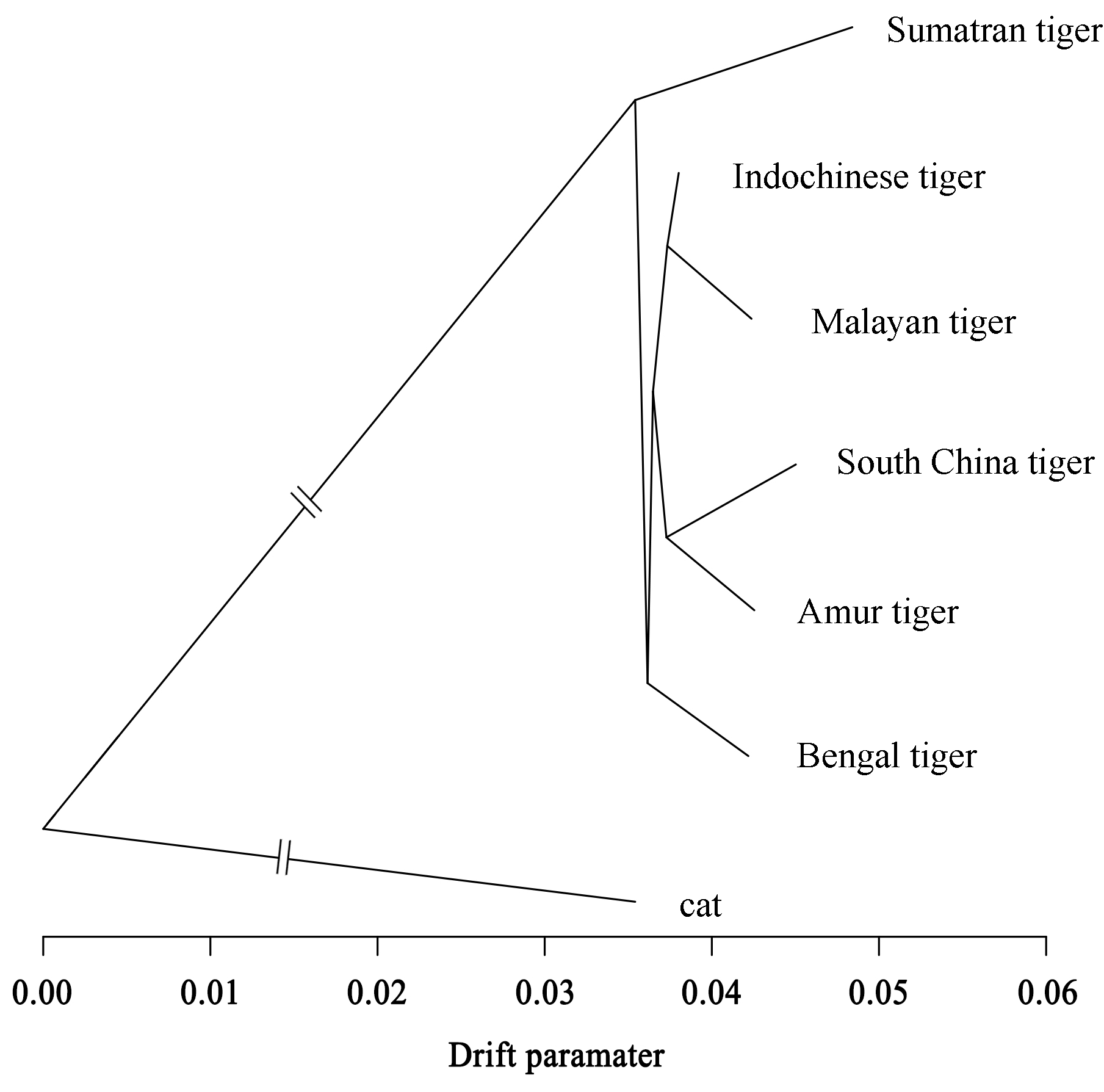


**Figure S11.** A maximum likelihood tree was built using TreeMix software with whole-genome sequencing data of six tiger subspecies and cat.
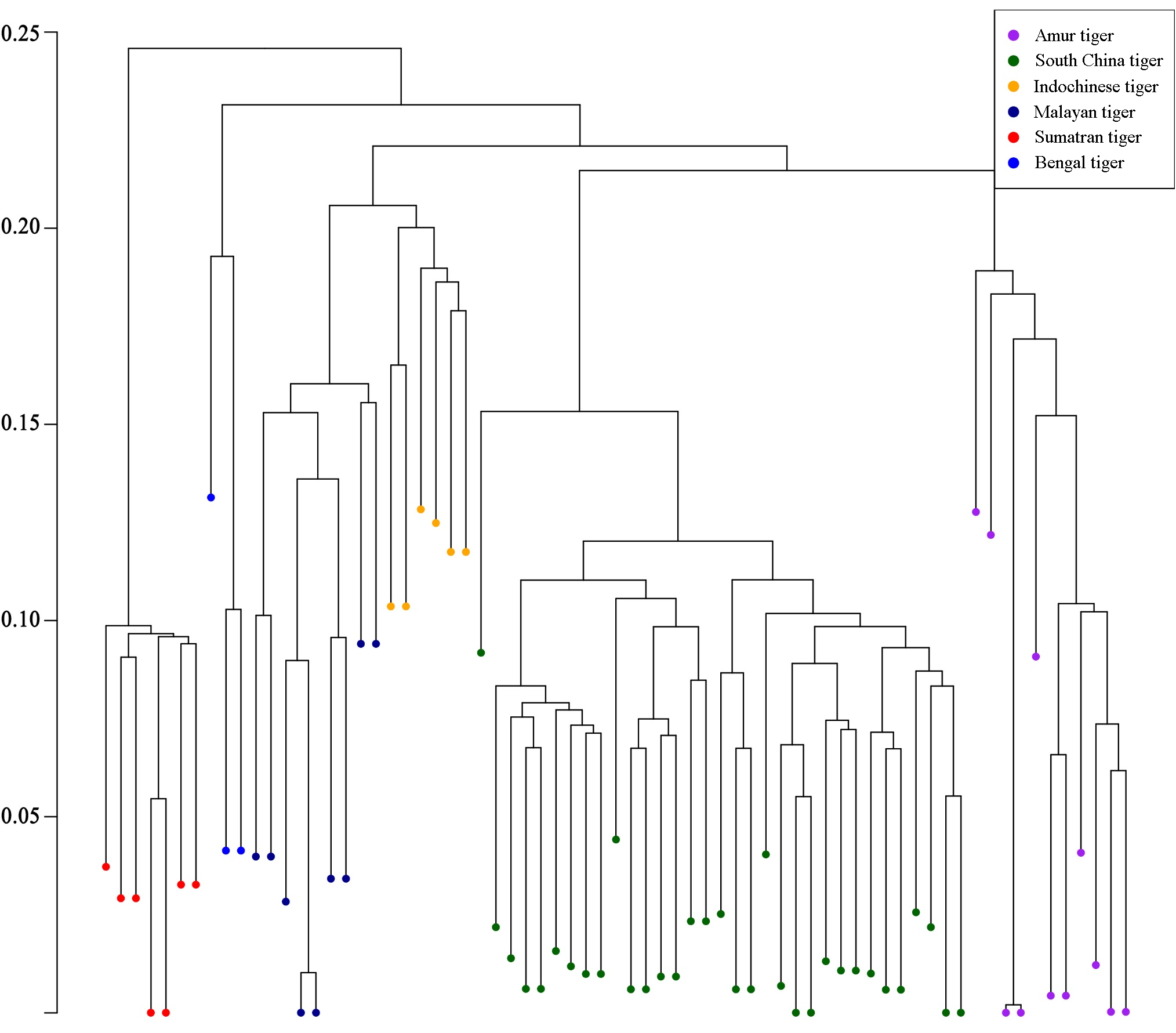


**Figure S12.** Cluster analysis based on the matrix of genome-wide identity-by-state (IBS) pairwise distances between six tiger subspecies determined by a permutation score.


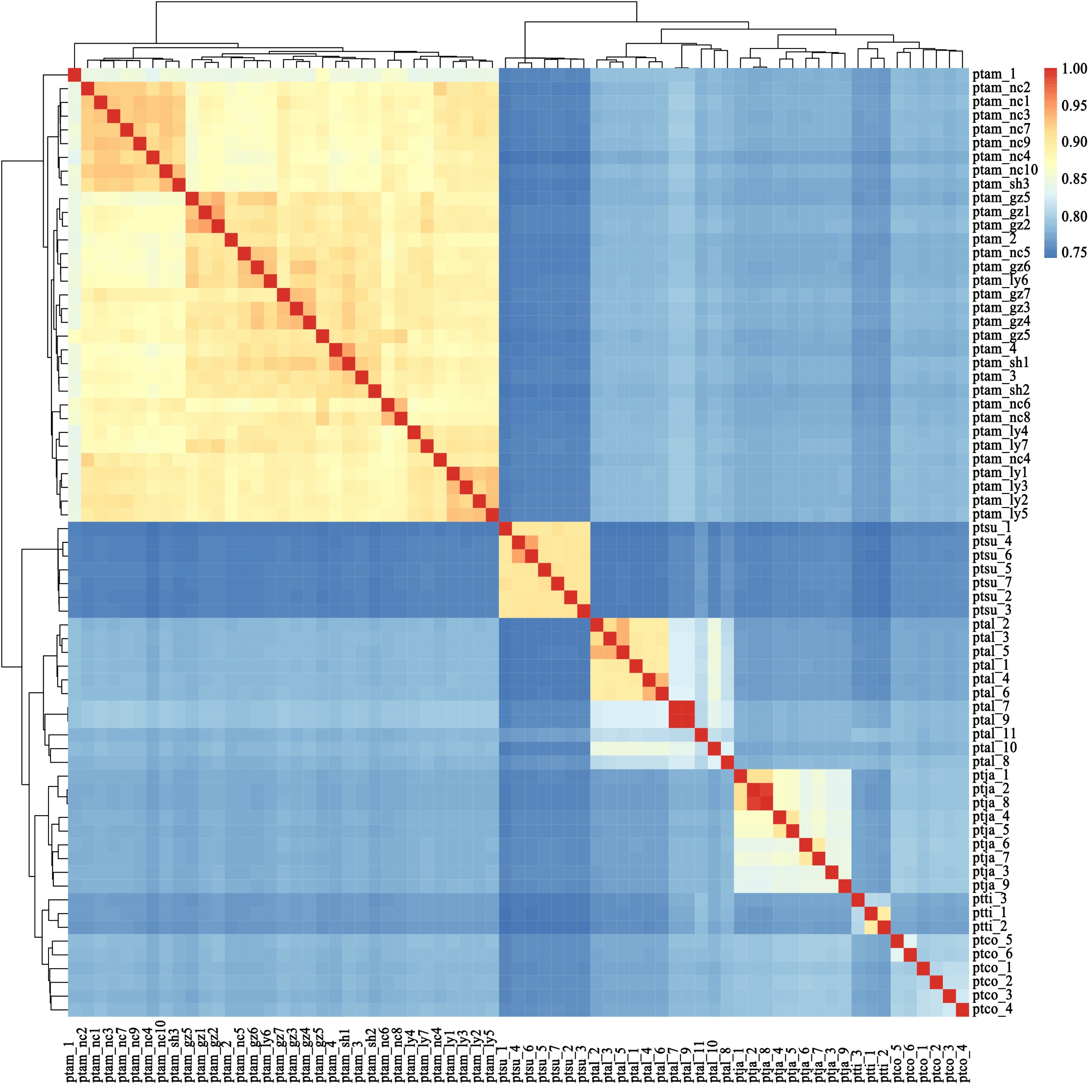


**Figure S13.** Analysis of genome-wide average identity-by-state (IBS) pairwise identities between six tiger subspecies.

**
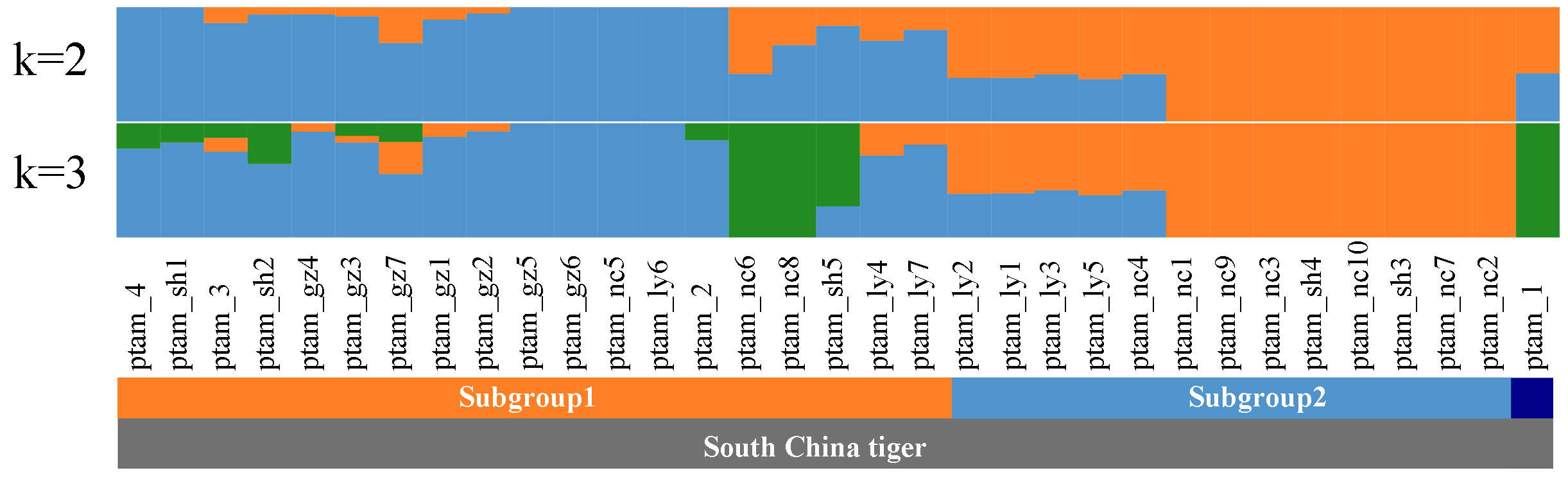
**

**Figure S14.** Population genetic structure of the South China tigers estimated by the ADMIXTURE.


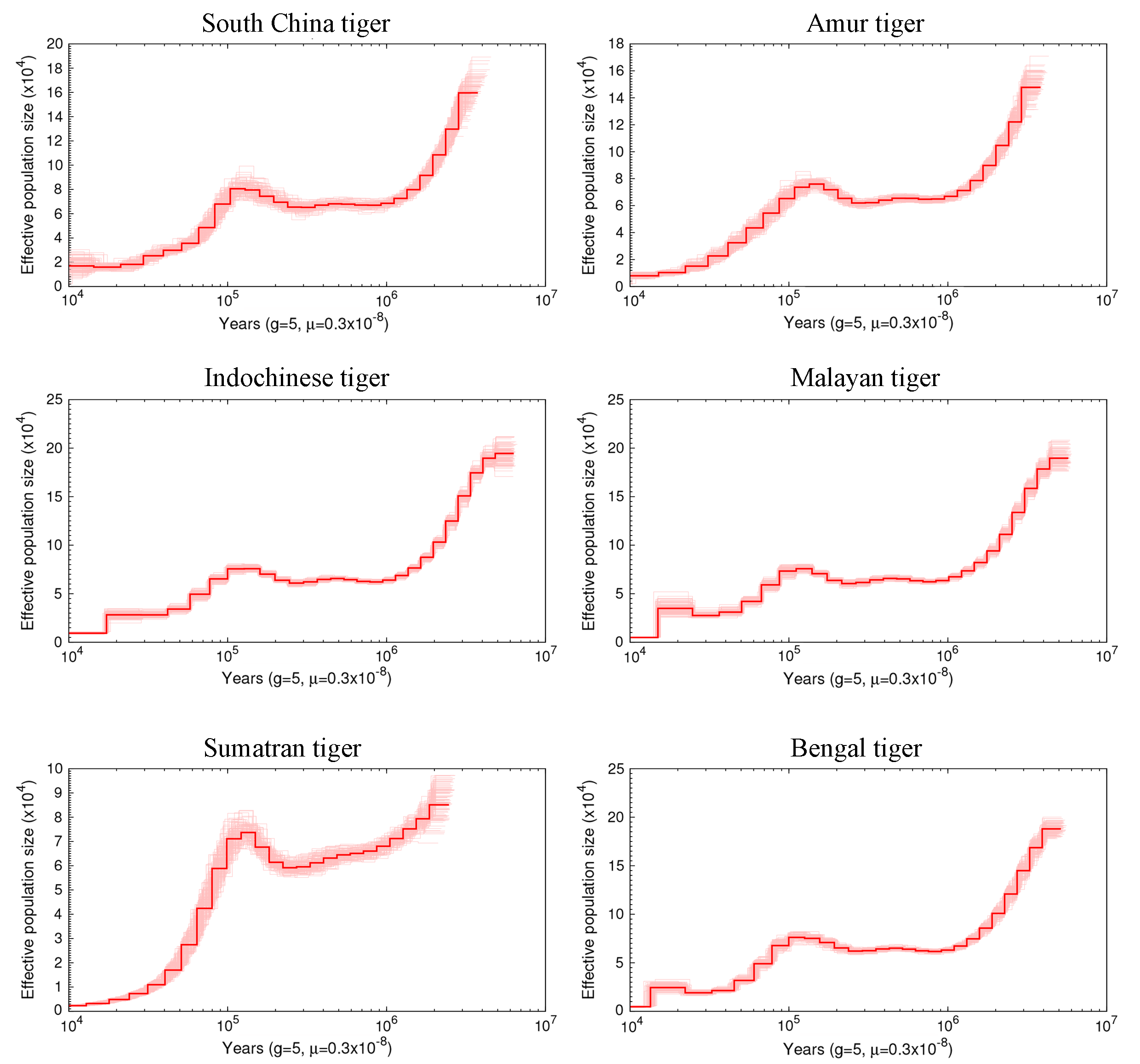


**Figure S15.** Demographic histories of tiger subspecies. PSMC analysis shows the change in effective population size over time. The dash lines represent 100 bootstraps. The representative individuals sequenced at a high read coverage were selected for each graph, South China tiger (ptam_4, mean 25.38×), Amur tiger (ptal_1, mean 14.76×), Indochinese tiger (ptco_2, mean 13.98×), Malayan tiger (ptja_7, mean 13.13×), Bengal tiger (ptti_3, mean 13.79×), Sumatran tiger (ptsu_1, mean 13.41×).

**
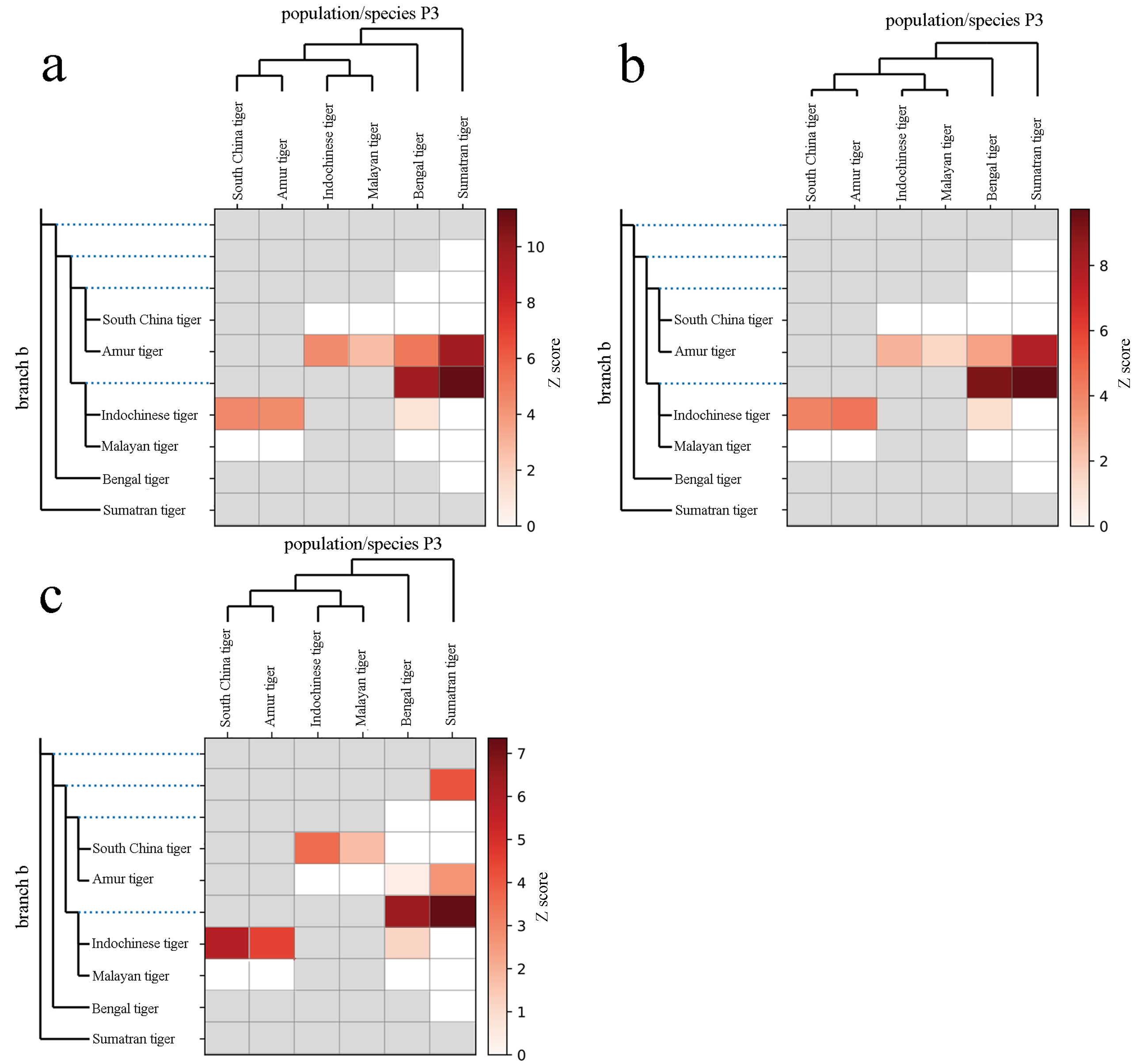
**

**Figure S16**. The results of Dsuite. Heatmap showing statistical support for introgression between pairs of tiger subspecies. Cells in the heatmap indicate the pairwise Z score values between the branch b identified on the expanded tree on the Y axis (relative to its sister branch) and the taxa P3 identified on the X-axis. The grey color indicates the none. (a) Result of Fbranch for South China tiger (subgroup1) with the other tiger subspecies populations. (b) Result of Fbranch for South China tiger (subgroup2) with the other tiger subspecies populations. (c) Result of Fbranch for South China tiger (ptam1 individual) with the other tiger subspecies populations.


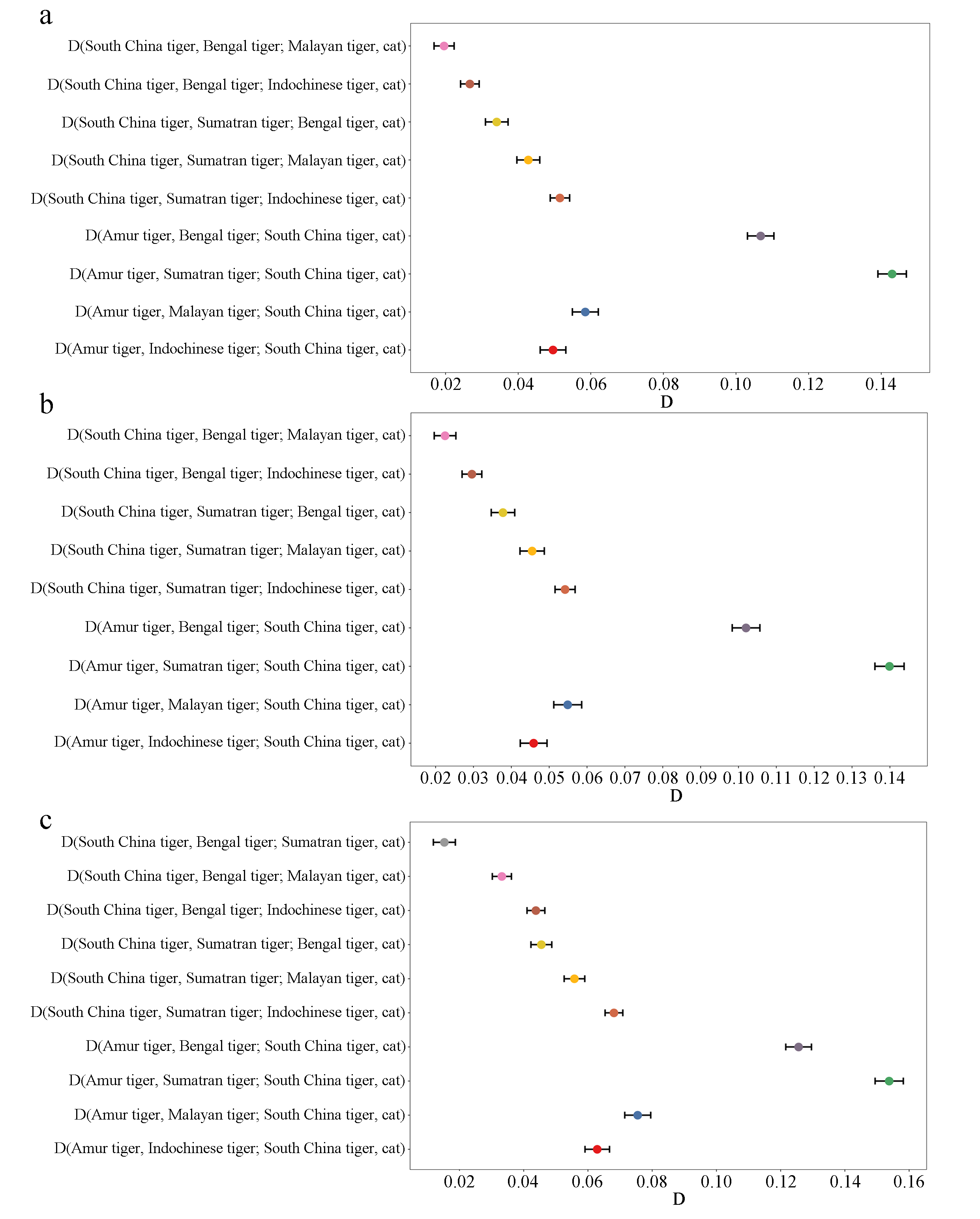


**Figure S17.** The graph of ABBA-BABA test. The values of D(pop1, pop2; pop3, pop4) ＞0 indicates that there are gene flows between pop1 and pop3. Here, only the introgression occurred in South China tiger will be shown. (a) D-statistic for South China tiger (subgroup1) with other tiger subspecies. (b) D-statistic for South China tiger (subgroup2) with other tiger subspecies. (c) D-statistic for South China tiger (ptam1 individual) with other tiger subspecies.


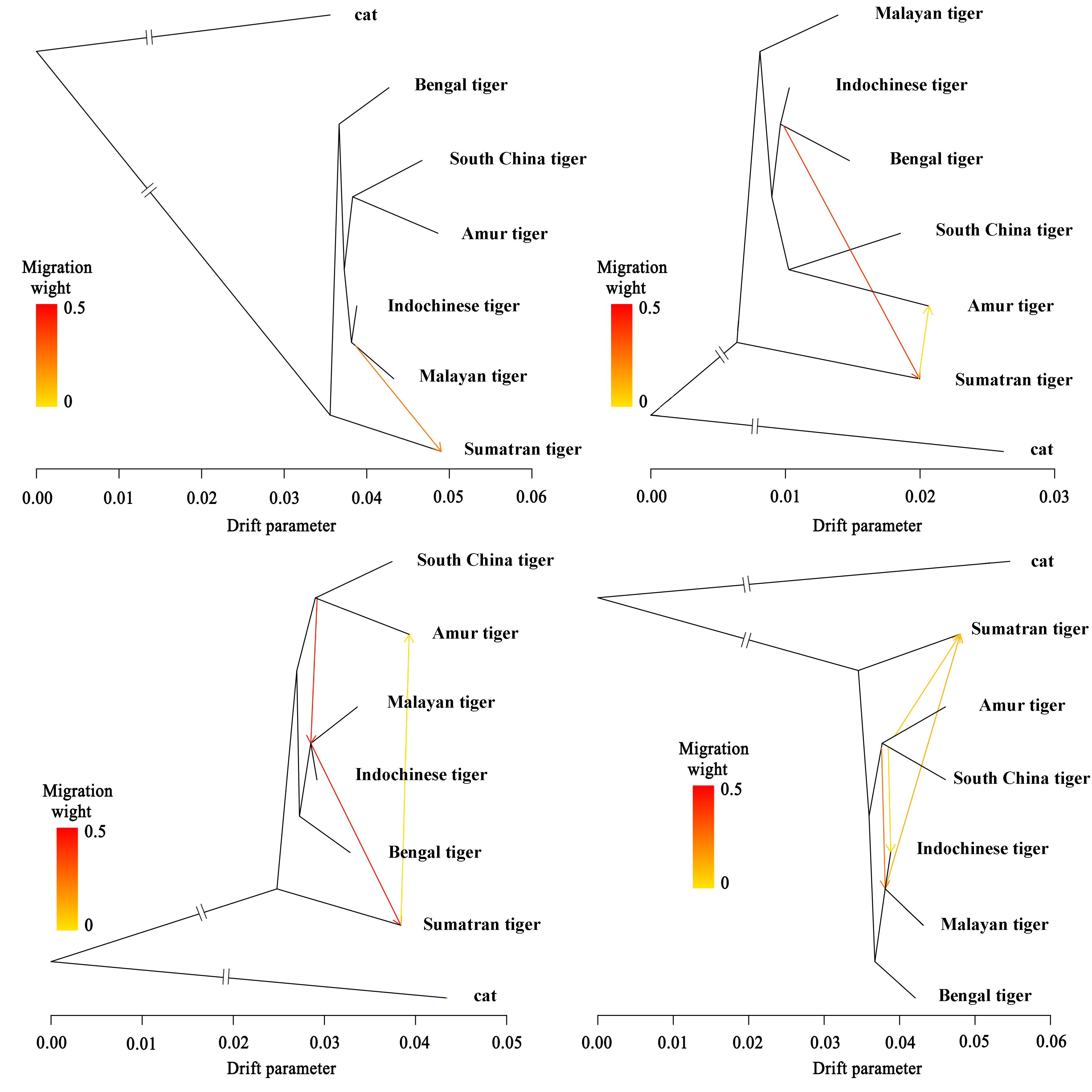


**Figure S18.** Plot of inferred introgression between the South China tiger subgroup1 and other tiger subspecies populations detected by the TreeMix method. The scale bar shows 10 times the average standard error of the entries in the sample covariance matrix.


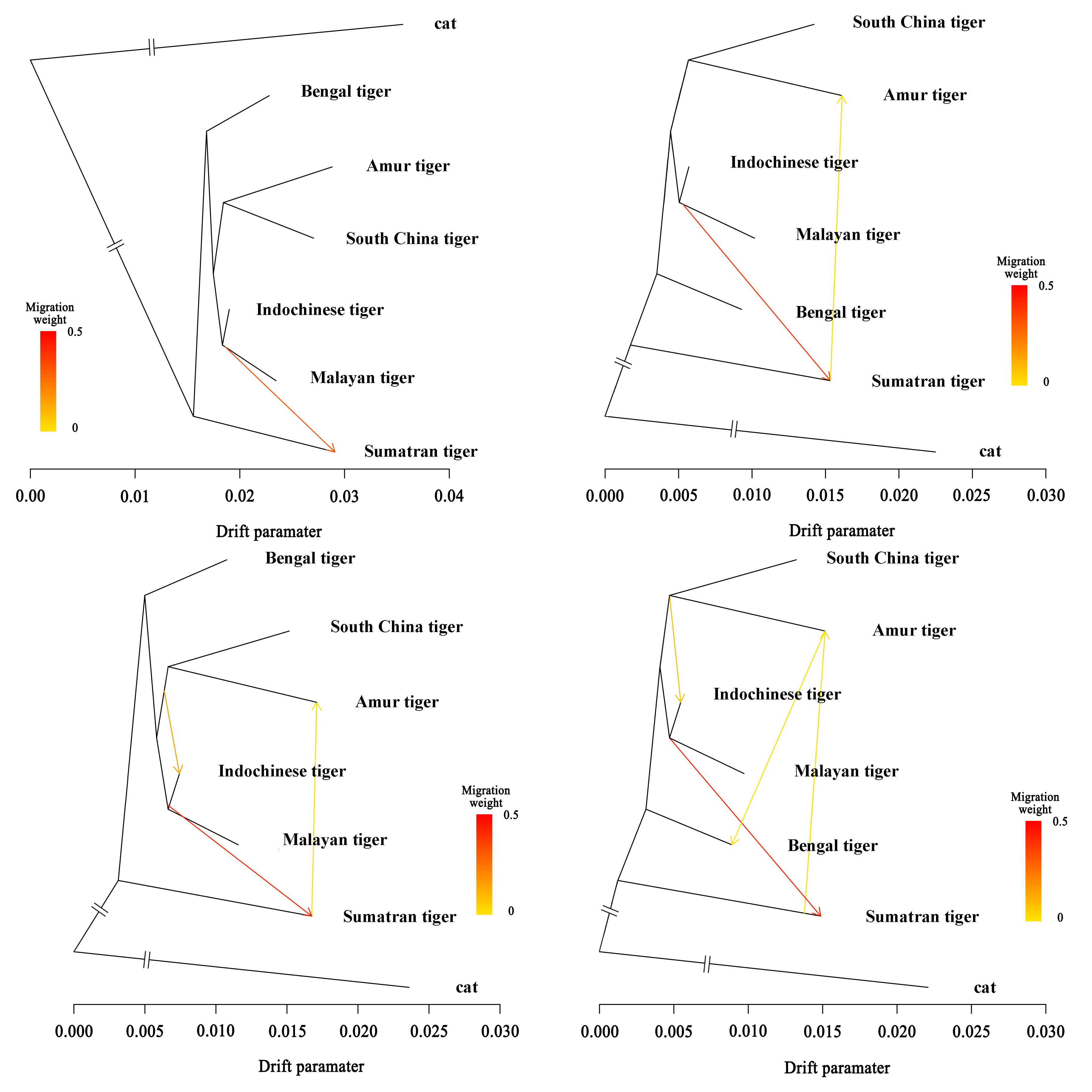


**Figure S19.** Plot of inferred introgression between the South China tiger subgroup2 and other tiger subspecies populations detected by the TreeMix method. The scale bar shows 10 times the average standard error of the entries in the sample covariance matrix.

**
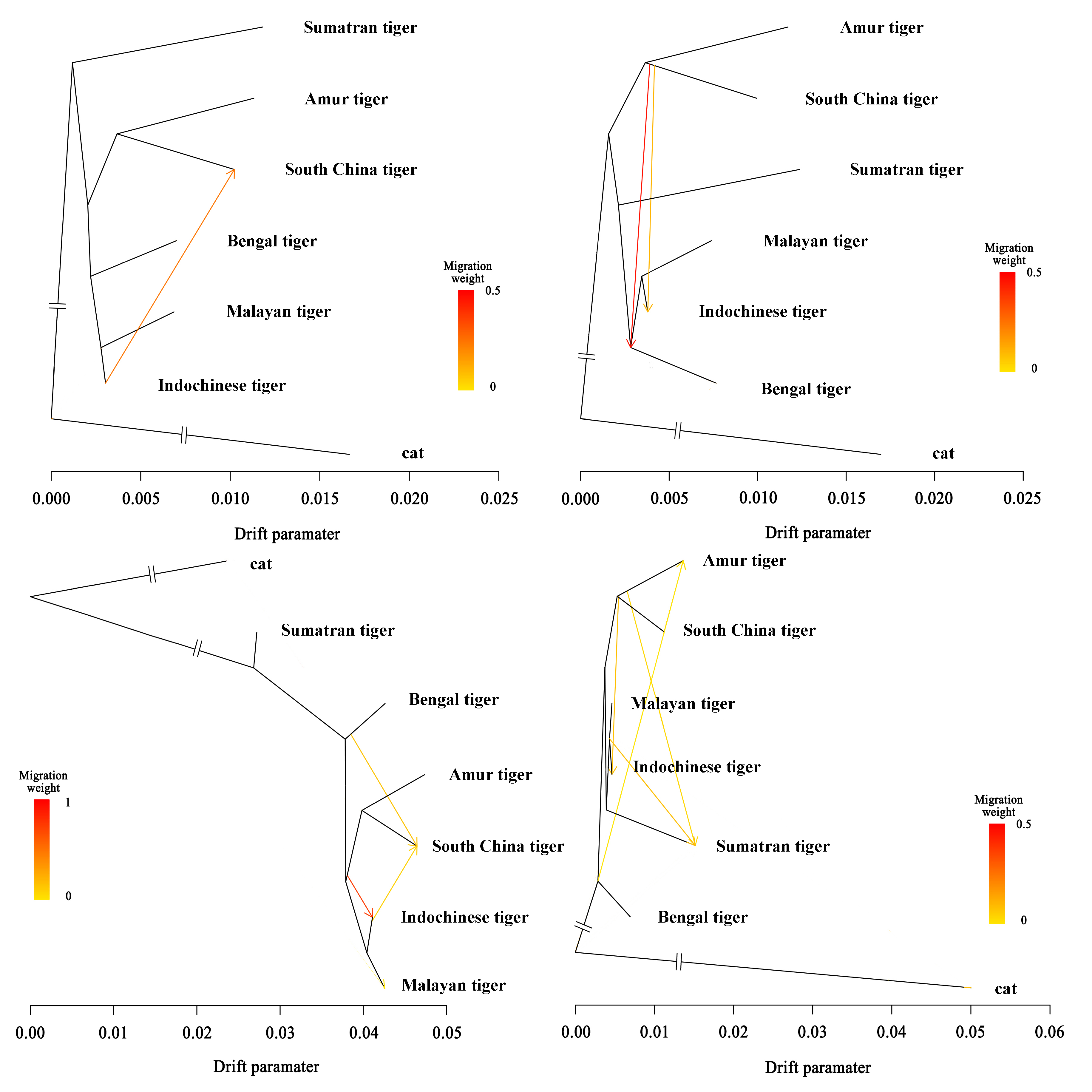
**

**Figure S20.** Plot of inferred introgression between the South China tiger ptam_1 individual and other tiger subspecies populations detected by the TreeMix method. The scale bar shows 10 times the average standard error of the entries in the sample covariance matrix.


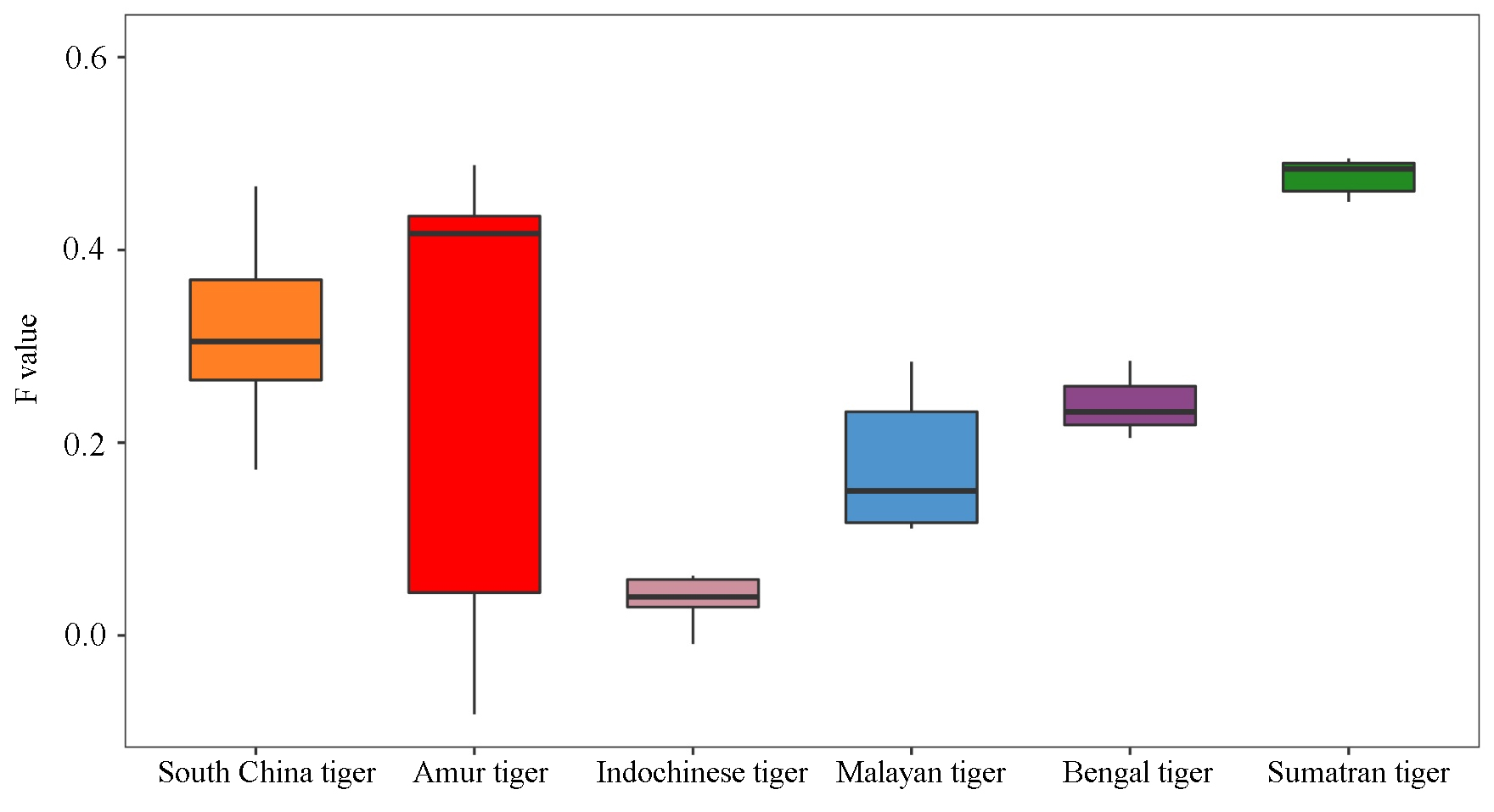


**Figure S21.** Genomic inbreeding coefficients *F*_H_ in each tiger subspecies populations.


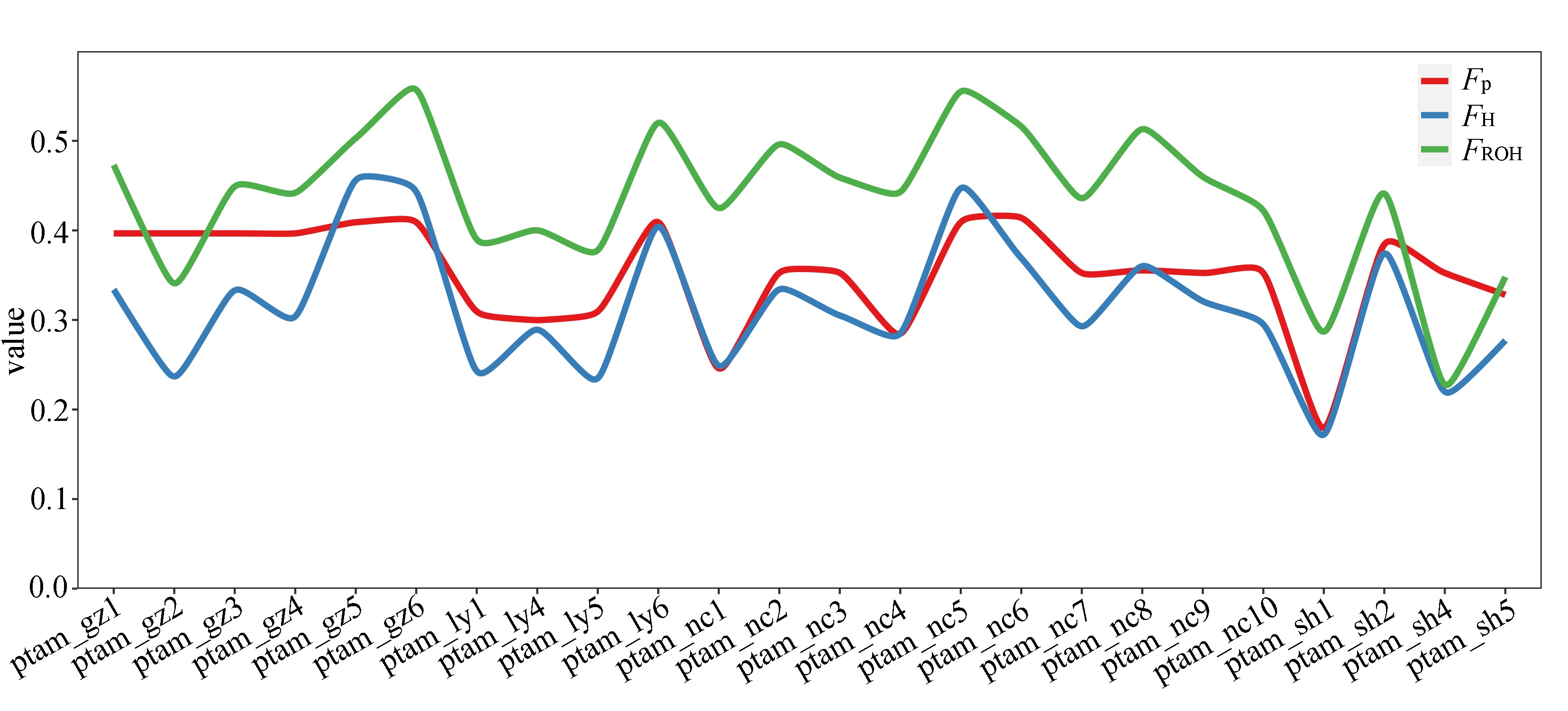


**Figure S22**. The plot of three inbreeding coefficients (*F*_P_, *F*_H_ and *F*_ROH_) of the South China tiger.


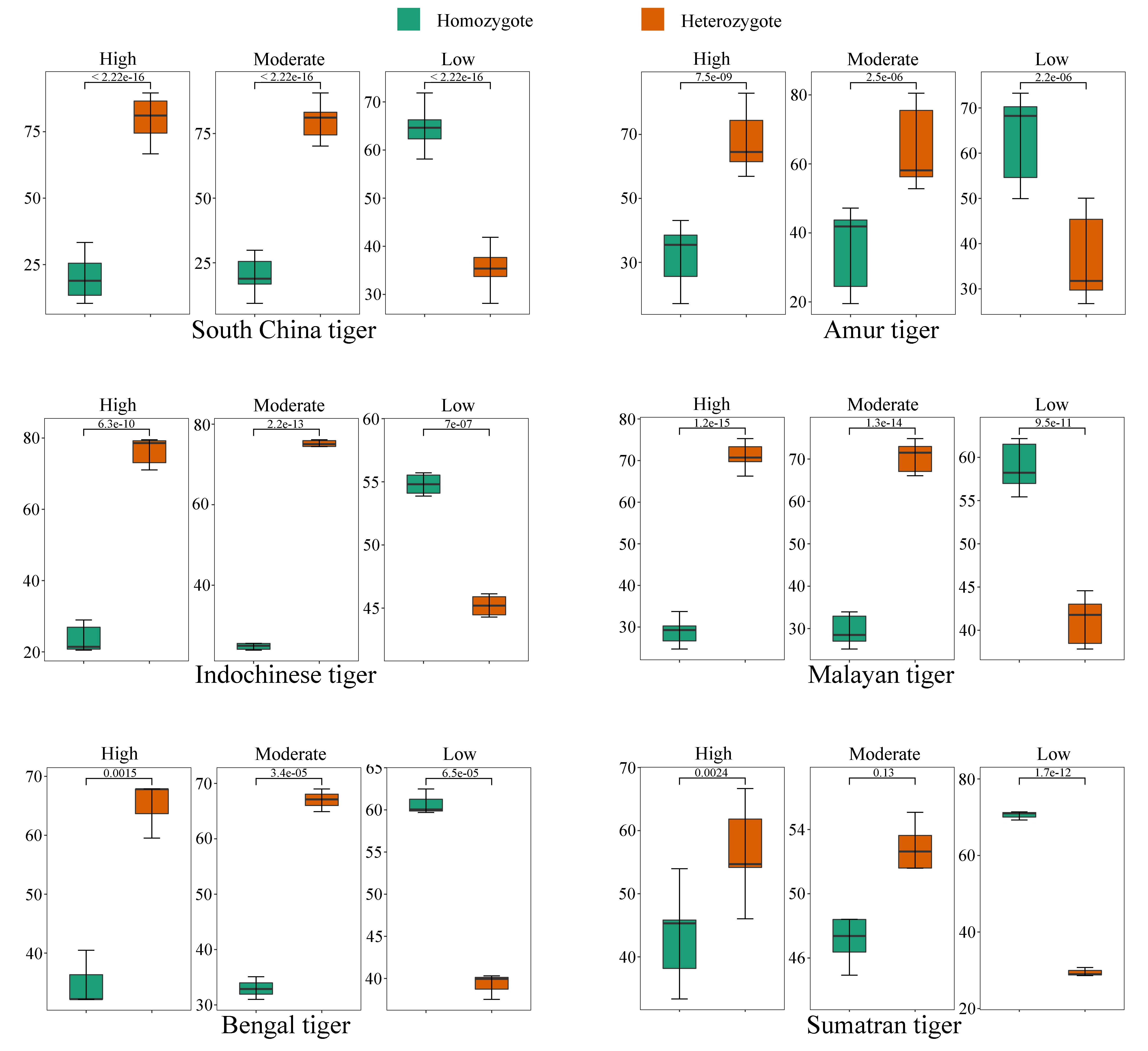


**Figure S23.** Comparison of homozygote and heterozygote percentage of per mutation category among six tiger subspecies. The significant value was calculated by *t*-test.
